# The discovery of 2-substituted phenol quinazolines as potent RET kinase inhibitors with improved KDR selectivity

**DOI:** 10.1016/j.ejmech.2016.01.039

**Published:** 2016-04-13

**Authors:** Rebecca Newton, Katherine A. Bowler, Emily M. Burns, Philip J. Chapman, Emma E. Fairweather, Samantha J.R. Fritzl, Kristin M. Goldberg, Niall M. Hamilton, Sarah V. Holt, Gemma V. Hopkins, Stuart D. Jones, Allan M. Jordan, Amanda J. Lyons, H. Nikki March, Neil Q. McDonald, Laura A. Maguire, Daniel P. Mould, Andrew G. Purkiss, Helen F. Small, Alexandra I.J. Stowell, Graeme J. Thomson, Ian D. Waddell, Bohdan Waszkowycz, Amanda J. Watson, Donald J. Ogilvie

**Affiliations:** a Cancer Research UK Manchester Institute, Drug Discovery Unit, University of Manchester, Wilmslow Road, Withington, Manchester, M20 4BX, England, UK; b Structural Biology Laboratory, Cancer Research UK London Research Institute, London, WC2A 3LY, England, UK; c Institute of Structural and Molecular Biology, Department of Biological Sciences, Birkbeck College, Malet Street, London WC1E 7HX, England, UK

**Keywords:** RET, Kinase, Quinazoline

## Abstract

Deregulation of the receptor tyrosine kinase RET has been implicated in medullary thyroid cancer, a small percentage of lung adenocarcinomas, endocrine-resistant breast cancer and pancreatic cancer. There are several clinically approved multi-kinase inhibitors that target RET as a secondary pharmacology but additional activities, most notably inhibition of KDR, lead to dose-limiting toxicities. There is, therefore, a clinical need for more specific RET kinase inhibitors. Herein we report our efforts towards identifying a potent and selective RET inhibitor using vandetanib **1** as the starting point for structure-based drug design. Phenolic anilinoquinazolines exemplified by **6** showed improved affinities towards RET but, unsurprisingly, suffered from high metabolic clearance. Efforts to mitigate the metabolic liability of the phenol led to the discovery that a flanking substituent not only improved the hepatocyte stability, but could also impart a significant gain in selectivity. This culminated in the identification of **36**; a potent RET inhibitor with much improved selectivity against KDR.

## Highlights

• Novel anilinoquinazoline RET inhibitors have been prepared.
• Phenolic moieties display enhanced potency but decreased metabolic stability.
• Substitution patterns adjacent to the phenol improve metabolic stability.
• These substituents deliver unanticipated improvements in selectivity.
• Improvements in selectivity are explained through X-ray crystallographic studies.

## Graphical abstract

**Figure:**
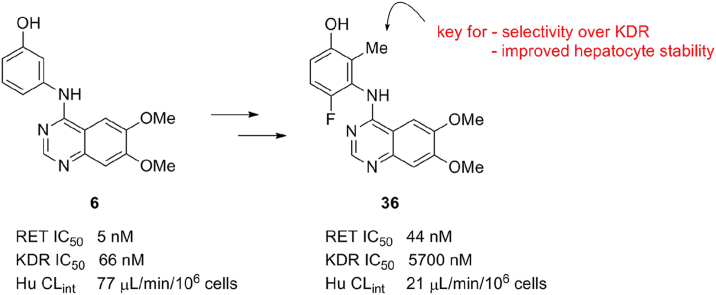

## 1 Introduction

**Fig. 1:**
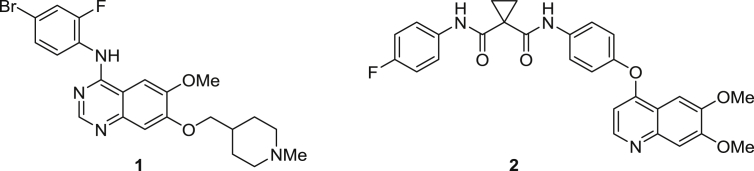
Multi-target kinase inhibitors which inhibit RET.

RET (REarranged during Transfection) is a receptor tyrosine kinase (RTK) that is required for normal development, maturation and maintenance of several tissues and cell types [1]. Gain of function mutations in RET are implicated in several human cancers, e.g. medullary thyroid cancer (MTC) and lung adenocarcinoma (LAD). The identification of these mutations and rearrangements in RET which lead to constitutive activation, together with convincing preclinical data validating RET as a classical oncogene, make this kinase an attractive target for cancer therapy. At present, there are no known specific RET inhibitors in clinical development, although many potent inhibitors of RET have been opportunistically identified through selectivity profiling of compounds initially designed to target other RTKs. The small molecule inhibitors vandetanib **1** and cabozantinib **2** (Fig. 1) exemplify this approach.

Although both have been approved for the treatment of advanced metastatic MTC [2], [3] and are undergoing Phase II trials in LAD [4], [5], RET inhibition is a secondary pharmacology of these drugs, which were initially developed as inhibitors of other receptor tyrosine kinases. Both agents target KDR (VEGFR2), whilst **1** has additional activity versus EGFR and **2** versus MET. Although it is possible that KDR activity may contribute to their clinical efficacy [6], the results of a large Phase III trial of **1** [2] showed a significantly better hazard ratio for RET-positive patients compared with RET-negative, suggesting that its efficacy is strongly related to its RET inhibitory activity. The EGFR activity of **1** is unlikely to significantly contribute to its efficacy since it has been demonstrated that selective inhibition of EGFR with gefitinib did not yield clinical responses in MTC [7]. Significant toxicity (e.g. rash, diarrhoea, hypertension) resulting from inhibition of these off-target kinases, particularly KDR, compromises the use of **1** and **2** in clinical settings [8]. Thus, there is a clear need for selective RET inhibitors which do not display the toxicities associated with the current treatments and enable more potent and sustained inhibition of RET signaling. These agents may offer greater clinical benefit for patients with RET mutant cancers and widen the scope for the clinical use of RET inhibitors. Although the initial clinical line of sight for this target was MTC, recent identification of RET fusions (e.g. KIF5B-RET and CCDC6-RET) present in approximately 1% of LAD patient samples offer an important disease segment in which a specific RET inhibitor would also offer clinical benefit. Additionally, RET has also recently been implicated in the progression of both breast and pancreatic tumours [9].

Our initial focus was to identify novel, low molecular weight, ATP-competitive inhibitors of the RET kinase domain with improved selectivity for RET against KDR in cellular assays, relative to **1**. The availability of high quality X-ray crystal structures of RET and KDR in the public domain enabled us to pursue a structure-guided medicinal chemistry approach to design and optimise novel and selective inhibitors. Hit identification employed a focused medicinal and computational chemistry programme to explore structure-activity relationships (SAR) around known RET scaffolds and to determine whether selectivity could be improved by targeting regions of the binding site that differ in sequence between RET and KDR. Although comparison of X-ray crystal structures of RET and KDR revealed that the ATP-binding pockets are generally very similar in structure, we hypothesized that there was potential to improve selectivity by targeting a number of specific residues readily accessible to the ligand. The anilinoquinazoline core of **1** was selected as a scaffold expected to impart good cellular activity and permeability, which allowed us to target binding site residues of interest using established medicinal chemistry methods.

**Fig. 2:**
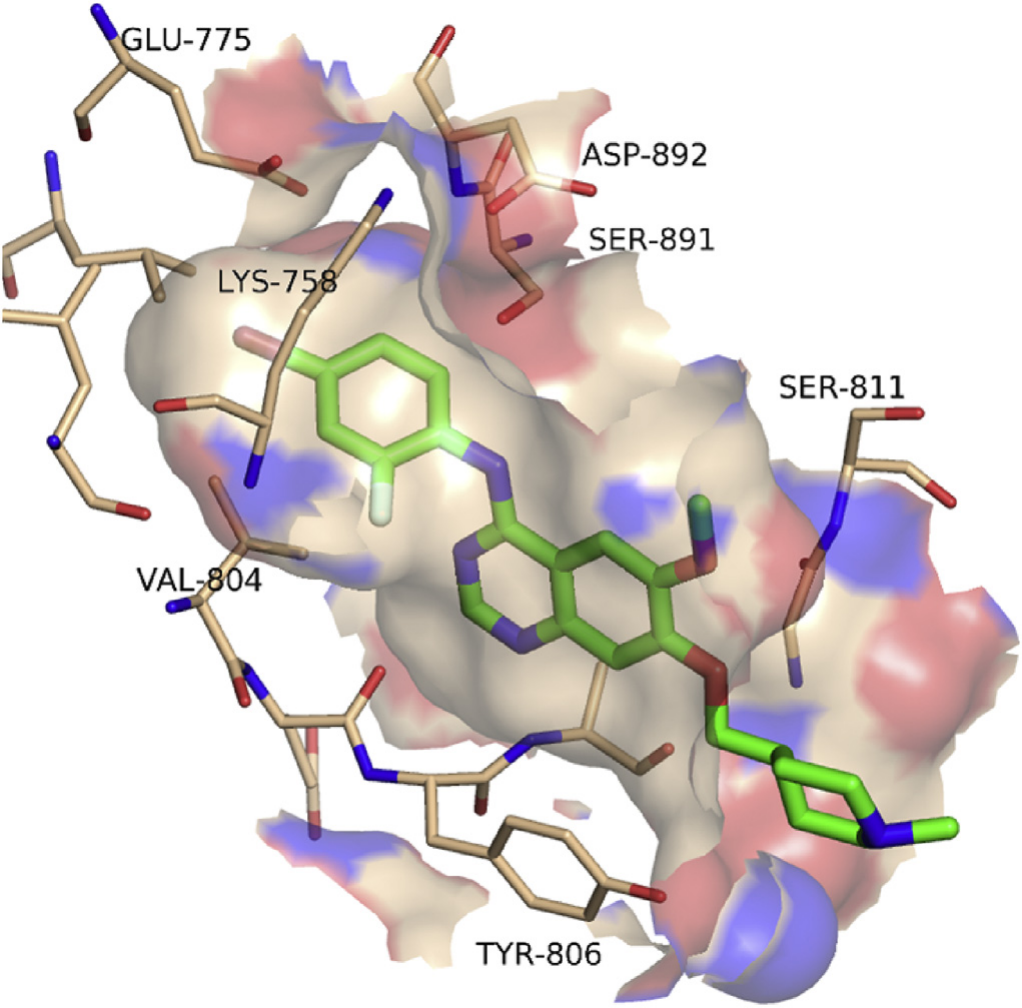
Location of key amino acids within the ATP-binding site of RET, illustrated using the X-ray structure of vandetanib (PDB code 2IVU). Amino acids that differ between RET and KDR include Ser891 (KDR Cys1045), Ser811 (KDR Asn923) and Tyr806 (KDR Phe918). Figure prepared using the Pymol Molecular Graphics System (Schrödinger, LLC, New York, NY).

The binding mode of **1** to the RET kinase domain has been reported previously [10]. The quinazoline core binds to the hinge region (Glu805-Ala807), with the anilino ring enclosed within the hydrophobic pocket between the gatekeeper residue Val804 and the catalytic lysine Lys758 (Fig. 2). As the gatekeeper pocket is well known to contribute to the affinity and selectivity of many series of kinase inhibitors, we decided to focus the initial optimization of the quinazoline series on exploring the SAR around the anilino ring, with a view to identifying substituents that improved selectivity towards RET. Given that the 6 and 7 positions on the quinazoline ring point primarily towards solvent, we expected these substituents to have a less pronounced role on selectivity, and hence fixed both positions as methoxy groups for the initial round of optimization.

## 2 Chemistry

### 2.1 Synthesis of anilinoquinazolines

**Scheme 1:**
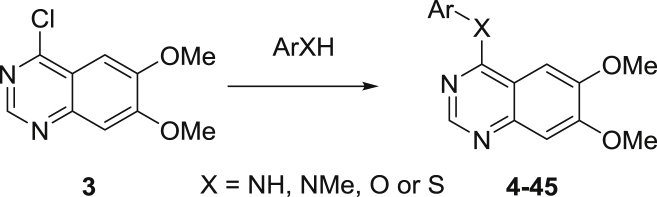
Synthesis of quinazolines.

Quinazolines **4**–**45** were prepared using the commercially available 6,7-bismethoxychloroquinazoline **3** as a simplified analogue of **1**, as illustrated in Scheme 1.

### 2.2 Synthesis of key intermediates

**Scheme 2:**
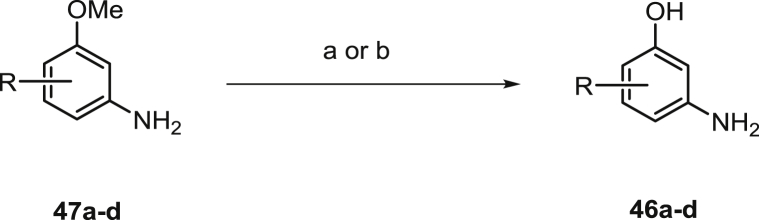
Synthesis of anilinophenols via demethylation. ^a^Reagents and conditions: (a) BBr_3_, DCM, -78 °C or (b) pyridinium chloride, 150 °C.

**Scheme 3:**
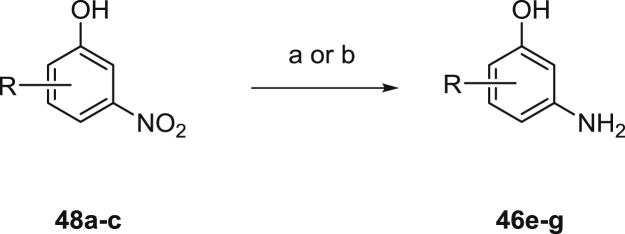
Synthesis of anilinophenols via nitro reduction. ^a^Reagents and conditions: (a) Pd/C, H_2_, EtOH or (b) Fe, AcOH, EtOH.

**Scheme 4:**
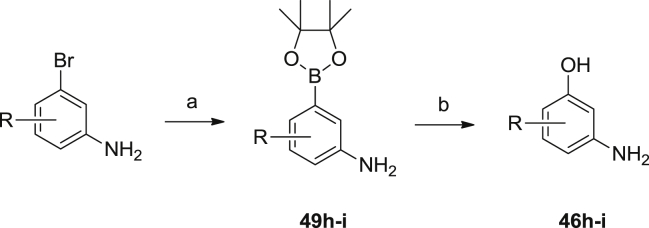
Synthesis of anilinophenols via boronate oxidation. ^a^Reagents and conditions: (a) Pd(dppf)Cl_2_·DCM, bis(pinacolato)diboron, 1,4-dioxane; (b) sodium perborate, THF, water.

**Scheme 5:**
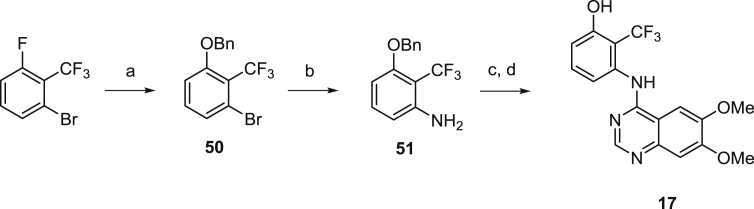
Synthesis of a benzyl-protected anilinophenol. ^a^Reagents and conditions: (a) BnOH, NaH, DMF; (b) (i) benzophenoneimine, Pd(OAc)_2_, BINAP, Cs_2_CO_3_, 1,4-dioxane; (ii) 2 M HCl, THF; (c) 3, IPA; (d) TFA.

**Scheme 6:**
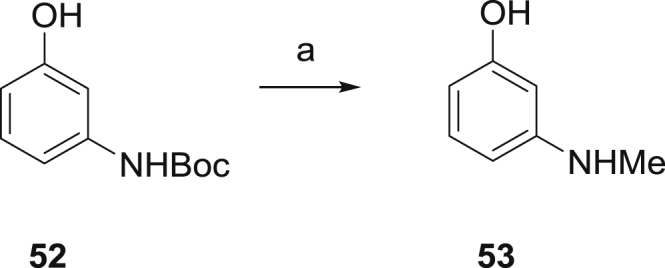
Synthesis of 53. ^a^Reagents and conditions: (a) LiAlH_4_, THF.

Many of the required nucleophiles were commercially available; the remainder was synthesized using known or modified chemistry. The routes used to prepare anilines **46a**–**i** are summarized in Scheme 2, Scheme 3, Scheme 4, Scheme 5, Scheme 6. Common precursors to the desired anilinophenols were the requisite methyl ethers **47a-d** or nitro compounds **48a**–**c**, which could be demethylated or reduced using standard procedures as shown in Scheme 2, Scheme 3.

Alternative precursors to the anilinophenols were the corresponding bromides. These could be converted to the boronate esters **49h**–**i**, then oxidized to the required phenols **46h**–**i** as shown in Scheme 4.

Attempts to prepare 3-amino-2-(trifluoromethyl)phenol were unsuccessful. Instead, benzyl ether **50** was prepared from commercial starting material then converted to aniline **51**. This was coupled directly with **3**, then deprotected to return the desired quinazoline **17** as shown in Scheme 5.

*N*-Methylated phenol **53** was prepared by reduction of the corresponding *N*-Boc material **52** (Scheme 6).

## 3 Results and discussion

### 3.1 Biochemical evaluation

Our initial aim was to prepare to number of anilinoquinazolines to explore the affinity and selectivity towards RET as compared to **1** [11], [12]. The biochemical data for selected compounds are shown in Table 1. First, we ascertained that switching from the more decorated quinazoline core present in **1** to the bis-methoxy scaffold present in **4** showed only a 2-fold drop in affinity (data for **1** not shown). Therefore, we opted to use this simplified scaffold for further exploration, given it was commercially available. Of the first swathe of approximately 30 simple anilinoquinazolines prepared, the most interesting observation was that phenol **6** resulted in a significant gain in affinity towards RET. The same level of affinity was not maintained in the isomeric compounds **5** and **7**, or for the analogous aniline **8** or the corresponding methoxy ether **9**.

### 3.2 Structural considerations

**Fig. 3:**
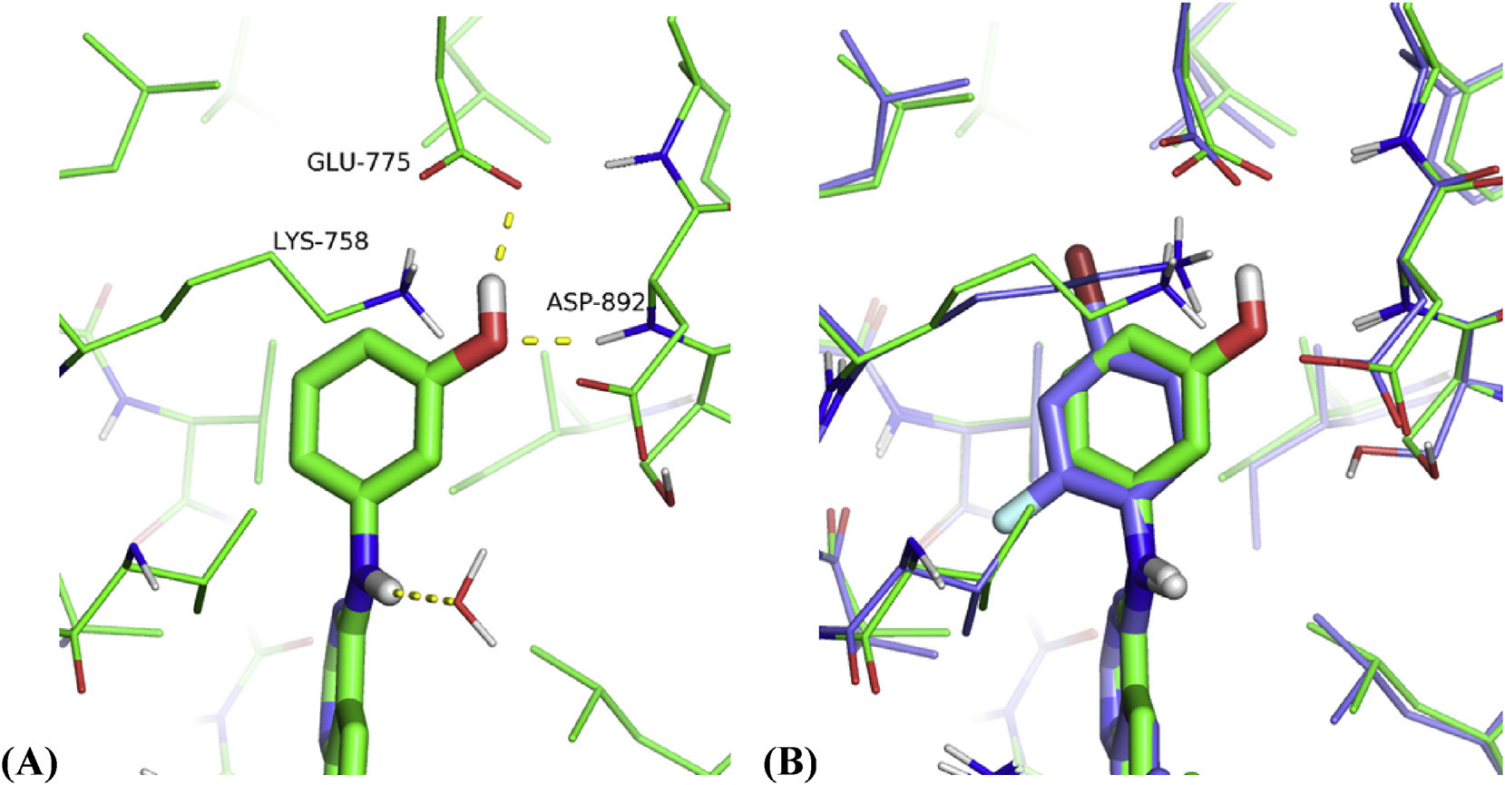
Detail of phenol interactions in the gatekeeper pocket of RET X-ray structures. (A) Crystal structure of compound **6** bound to RET kinase domain, highlighting hydrogen-bonding interactions to Glu775 and Asp892N–H (PDB 5AMN). (B) Comparison of compound **6** with the published structure of 1 (PDB 2IVU, carbon atoms in purple). Hydrogen atoms modelled using Maestro and figure prepared using the Pymol Molecular Graphics System (Schrödinger, LLC, New York, NY). (For interpretation of the references to colour in this figure legend, the reader is referred to the web version of this article.)

The boost in RET affinity from the R^2^ hydroxyl group may be rationalised by consideration of the hydrogen bonding contacts formed in the gatekeeper pocket. Initial modelling of **6**, subsequently confirmed by the determination of the X-ray structure bound to RET (Fig. 3), highlighted a pair of hydrogen bonds from the phenol to the side-chain of Glu775 (the conserved glutamate located on the αC-helix) and the backbone NH of Asp892 (from the conserved DFG motif). It is likely that this specific network of hydrogen bonds is responsible for the gain in affinity and the tight SAR for the R^2^ hydroxyl, with alternative polar R^2^ substituents or hydroxyls in the R^1^ or R^3^ positions all demonstrating reduced affinity

Comparison of the X-ray structures of **6** and **1** reveals that the ligands share very similar binding modes. The anilino ring of **6** binds between the gatekeeper Val804 and the catalytic lysine Lys758 and, in comparison to **1**, shows a small rotation towards Asp892, with minor re-orientation of neighbouring side-chains consistent with the formation of hydrogen-bonding interactions between the phenol and Glu775/Asp892. The most substantial structural change is the displacement of the phosphate-binding loop away from the ATP binding site in the X-ray structure of **6**; this may be a consequence of a degree of induced fit in the ligand binding site or may simply reflect the inherent flexibility of this loop. Comparison with X-ray structures of **6** bound to other kinases, e.g. to CDK2, CDPK1 and TTBK1 (PDB codes 1DI8, 3NYV and 4BTK, respectively), reveal a variety of different conformations and hydrogen bonding interactions for the phenol moiety, distinct from that observed for **6** bound to RET. Thus it should be possible to modulate the selectivity of this series by appropriate substitution around the anilino ring.

### 3.3 Mitigation of DMPK concerns and delivery of unanticipated selectivity

The presence of a phenol was some cause for concern given it was anticipated to undergo phase II metabolism. Measurement of in vitro DMPK properties (data not shown) of the initial compound **6** showed that solubility and CYP inhibition were acceptable at this stage, although the permeability and efflux needed improvement. Observation of metabolism in microsomes, albeit to a 3-fold lesser extent than in hepatocytes, indicated phase I metabolism was occurring in addition to phase II.

In order to mitigate phase II metabolism, we explored further substitution on the phenolic aniline. Our goals here were two-fold. First, it was speculated that the presence of flanking substituents might attenuate the propensity of the phenol to undergo conjugation, thereby increasing hepatocyte stability. Second, it would allow a more general SAR exploration to determine what functionality could be tolerated around the phenyl ring.

We first prepared a number of mono-substituted anilinophenols. Most of these bore a flanking substituent either at the R^1^ or R^3^ position, primarily to investigate whether the hepatocyte stability could be mitigated through steric encumbrance. However, it soon became apparent that a suitable R^1^ substituent also had a considerable influence on KDR selectivity (**10**–**13**); albeit with some loss of RET affinity for **11**–**13**. This selectivity enhancement was less evident with fluorine (**14**), whereas larger substituents were not tolerated, as indicated by the large drop in RET affinity (**15**–**18**). Conversely, a flanking substituent at R^3^ (**19**–**22**) was tolerated with respect to RET affinity but selectivity was comparable to, or worse than, the unsubstituted phenol **6**. Although we anticipated that any improvement in hepatocyte stability would be most likely achieved by exploiting substitution at the flanking R^1^ or R^3^ positions, limited examples of substitution at the R^4^ or R^5^ position were also explored. Halogens were tolerated at R^4^ and R^5^ (**23**, **25**–**26**) but a methyl group resulted in a modest drop in affinity, especially at the R^4^ position (**24** and **27**).

In addition to these mono-substituted examples, we also prepared a number of di-substituted anilinophenols. Appropriately positioned halogens generally retained or enhanced affinity but selectivity was modest in the absence of a substituent larger than either H or F at the R^1^ position (**28**–**34**).

Clearly, incorporation of a suitable group at the R^1^ position was beneficial for selectivity, although affinity was somewhat diminished, especially when R^1^ was Me. Given that halogens, especially fluorine, at R^5^ had been seen to generally enhance affinity, it was encouraging to see that combining these two observations resulted in compounds which now exhibited both improved affinity and selectivity (**35**–**37**). When R^1^ was fixed as chloro, substituting at R^3^ with a methyl group was detrimental with regard to selectivity (compare **12** with **38**). Further substitution at R^5^ with chlorine (**39**) was tolerated whereas fluorine (**40**) appeared to be beneficial, both in terms of affinity and selectivity.

**Fig. 4:**
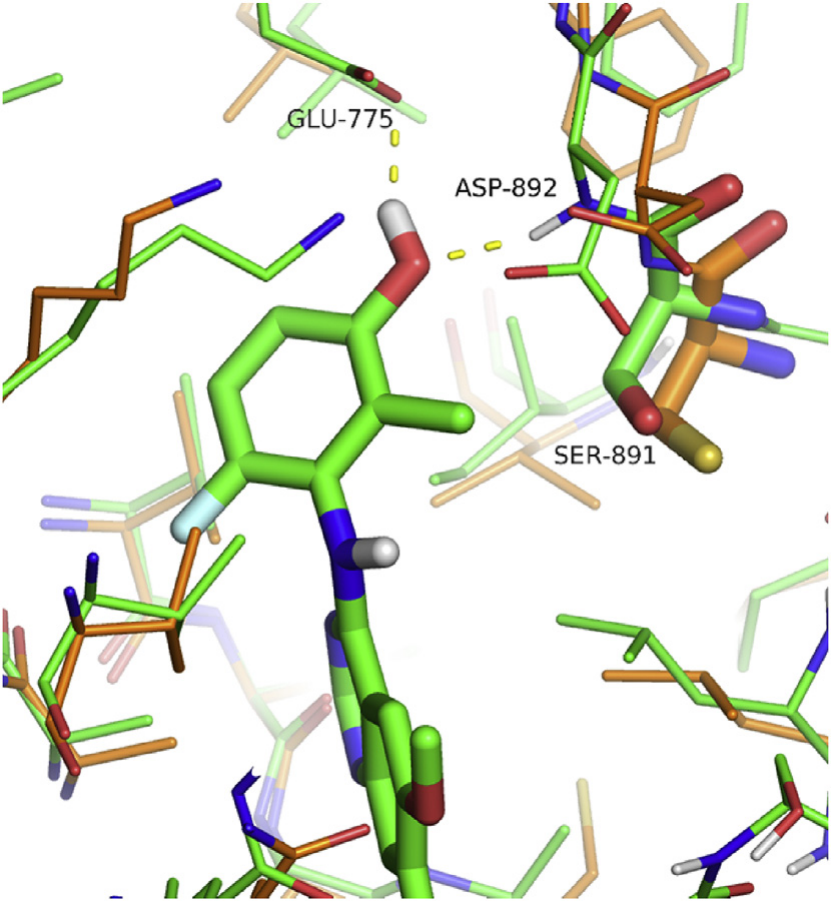
Compound **36** modelled in RET (green carbon atoms), compared with the aligned X-ray structure of KDR (PDB 3CJG, orange carbons). The R^1^ methyl group approaches close to the sidechain of Ser891 of RET, equivalent to the bulkier Cys1045 of KDR. (For interpretation of the references to colour in this figure legend, the reader is referred to the web version of this article.)

Modelling of this series in RET suggests that the R^1^ methyl substituent is positioned close to the side-chain of Ser891 (immediately preceding the DFG motif), and this potentially disfavoured contact could account for the observed reduction in RET affinity compared with **6** (Fig. 4). In KDR, Ser891 is replaced by a bulkier cysteine (Cys1045). Hence, although the R^1^ methyl is somewhat disfavoured in RET, there is a larger drop-off in affinity against KDR, leading to an improved selectivity profile towards RET overall.

### 3.4 Evaluation in cellular assays

A number of these compounds (selected on the basis of affinity and/or selectivity) were progressed into BaF3 cellular assays for RET and KDR, the results of which are shown in Table 2. Disappointingly, the affinity and selectivity observed for this phenolic series in the biochemical assay did not transfer well to the cellular context. Only 4 compounds (**25**, **26**, **28** and **30**) were <100 nM against RET in the cellular assay and only one compound (**40**) showed >10-fold selectivity versus KDR. In contrast, 17 compounds were ≤10 nM in the biochemical assay and 22 compounds showed >10-fold selectivity. For reasons not fully understood, the disconnect from the biochemical to cellular assay is much greater for RET than KDR, in effect compressing the selectivity margins, often from >100-fold in the biochemical assay to parity (or worse) in the cellular assay. Permeability was not believed to be the cause for the disparity as the observed reduction in affinity was not of the same magnitude for both RET and KDR in a matched cell line. The affinity for ATP in the biochemical assay is also unlikely to explain the difference in the reduction as the *K*_*m*_ values for both proteins (RET 9 μM and KDR 8 μM) were similar. Interestingly, the non-phenolic quinazoline **4** does not appear to suffer to the same extent.

The discrepancy between biochemical and cellular selectivity may be related to the binding mode of the R^2^ phenol. Modelling of **6** in various published X-ray structures of KDR suggested that the hydrogen bonding network around the phenol in RET may be less readily achievable in KDR. This is a consequence of KDR X-ray structures displaying a conformation in which the αC-helix is displaced away from the ATP binding site in comparison to RET. The associated movement of the conserved glutamate located on the αC-helix (equivalent to Glu775 in RET) would likely compromise the formation of the pair of hydrogen bonds to the phenol observed crystallographically in RET. This “αC-helix-out” motif is typically characteristic of an inactive kinase conformation. It may be that the difference between biochemical and cell selectivity profiles is related to different populations of active/inactive RET conformations under the different assay conditions. Thus, under the conditions of the biochemical assay, RET may exist predominantly in the active conformation characterized in the available X-ray structures, whereas in the cellular context there may be a larger population of inactive conformations, similar to the “αC-helix-out” conformation observed in KDR X-ray structures, to which binding of the phenol is less favoured. Regardless of the explanation for the observed differences, the decision was taken that the more physiologically relevant cellular data would drive subsequent progression of the project.

### 3.5 Investigation of alternate chemotypes

Different linkers were investigated as an alternative to the aniline but showed no improvement in comparison to **6**. As shown in Table 3, it can be seen that the *N*-methyl analogue **41** is significantly less potent than the NH-, O- and S-linked analogues (**6**, **42** and **43**). Although **42** and **43** retain biochemical affinity as compared to **6**, they too suffer from a disproportionate reduction in affinity against RET compared to KDR in the cell assay.

Despite the drop in affinity of the *N*-methylated analogue **41**, it retained some activity. Based upon this observation, the tethered compounds **44** and **45** were synthesized to test the hypothesis that these may deliver the same gain in selectivity as did the 2-substituted analogues described earlier. However, although this was observed biochemically for **44**, these biochemical activities again did not translate to cellular potency and selectivity, as shown in Table 4.

### 3.6 Further biological profiling

As mentioned previously, the initial interest in preparing phenolic anilines with flanking substituents was to mitigate the anticipated metabolic liability of the phenol. Table 5 shows the human hepatocyte stability data for selected compounds, ranked from most to least stable. Although compounds **10**, **11** and **12** benefitted from an improved selectivity profile compared to unsubstituted **6**, the R^1^ groups in these examples were detrimental to hepatocyte stability. Pleasingly, **13** did show the desired improvement in stability, albeit with a 20-fold reduction in affinity. Inclusion of fluorine at either the R^4^ or R^5^ positions went some way toward recovering the affinity but it can be seen that **36** is preferred over **35** with regard to hepatocyte stability. Interestingly, the isomer **30**, bearing the flanking substituent in the R^3^ position rather than the R1 position as in **36**, is significantly less stable. Unfortunately, **33**, **34** and **40** (the most promising compounds with regard to affinity and selectivity in the cell assay) all suffered from high clearance.

There does not appear to be a clear correlation between stability and phenol p*K*_a_, indicating other effects (e.g. steric influence of the flanking substituent or contributions from other substituents in the case of the di-substituted examples) are contributing to the observed clearance across the series. Looking at those compounds mono-substituted at R^1^ (**13**, **10**, **11** and **12**), only the Me-substituted compound **13** shows an improvement in clearance when compared to the unsubstituted phenol **6**. Although it is true that, for this particular set of compounds, a lower p*K*_a_ results in higher clearance, this is not observed in all cases. Isomeric pair **30** and **36** have identical calculated p*K*_a_ values yet differ only by the position of the flanking Me substituent, indicating that substitution with a Me group at the R^3^ position is detrimental to metabolic stability. Comparison of **30**, **35** and **36** shows the inclusion of F at R^5^ is beneficial, although the effect on p*K*_a_ is negligible, whereas inclusion of F at R^4^ resulted in a considerably lower phenol p*K*_a_ and higher clearance. Compounds containing a halogen at both R^1^ and R^5^ displayed lower clearances than those only substituted with a halogen at R^1^, despite similar p*K*_a_ values (compare **33**, **34**, **40**, **11** and **12**), again indicating that blocking R^5^ is beneficial. There does not appear to be any correlation between clearance and predicted log*P* (XLogP).

These compounds were also tested for non-specific cellular toxicity, and, with the possible exception of **30**, all were found to be devoid of non-specific toxicity in a wild-type BaF3 cell line, the parental cell line used to prepare the RET and KDR driven cell lines used in our routine screening assays. This pleasing result further suggests that the compounds display meaningful kinase selectivity in the cellular context and do not promiscuously inhibit off-target kinases responsible for cell proliferation and survival.

On the basis of these data, **36** was selected for further in vitro and in vivo pharmacokinetic assessment. In terms of metabolic stability, intrinsic clearance was higher in human hepatocytes than in human microsomes (CL_int_ 6.2 μL/min/mg), indicative of phase II metabolism. Metabolism was more rapid in mouse in both microsomes and hepatocytes (CL_int_ 28.2 μL/min/mg and 38.1 μL/min/10^6^ cells, respectively). In terms of physical properties, **36** showed good aqueous solubility (in excess of 100 μM) but only moderate permeability in Caco-2 cells (P_app_ 8.2 × 10^−6^ cm s^−1^, efflux ratio 4.9). Pharmacokinetics were measured in the mouse via intravenous and oral routes of administration. Total blood clearance was low (<10% LBF) and bioavailability was approximately 35%. Oral half-life was measured at approximately 2 h.

## 4 Conclusion

A structure-based drug design programme led to a series of phenolic anilinoquinazolines showing high affinity for RET in the biochemical context. Concern over the metabolic liability of phenol **6** prompted exploration of flanking substituents to attenuate the propensity of the phenol to undergo phase II metabolism. Pleasingly, incorporation of Me at R^1^ not only resulted in improved metabolic stability but also in an unexpected gain in selectivity over KDR, which could be rationalised by modelling. The improved selectivity was accompanied by some reduction in affinity but this could be recovered to some extent by inclusion of fluorine at the R^5^ position, resulting in **36**; a potent and selective RET inhibitor. However, for reasons not fully understood, the translation of biochemical potency to cellular potency was disproportionate when comparing RET and KDR, in effect compressing the apparent selectivity observed in the biochemical assay. Further efforts to improve both the cellular affinity and selectivity and the ADME properties of **36** are underway in our laboratory.

## 5 Experimental

### 5.1 Chemistry

All reagents obtained from commercial sources were used without further purification. Anhydrous solvents were obtained from the Sigma-Aldrich Chemical Co. Ltd. or Fisher Chemicals Ltd. and used without further drying. Solutions containing products were either passed through a hydrophobic frit or dried over anhydrous MgSO_4_ or Na_2_SO_4_, and filtered prior to evaporation of the solvent under reduced pressure. Thin layer chromatography (TLC) was conducted with 5 cm × 10 cm plates coated with Merck type 60 F_254_ silica gel to a thickness of 0.25 mm. Chromatography was performed on Biotage SNAP HP-Sil cartridges using a CombiFlash Companion machine. Proton (^1^H) NMR spectra were recorded on a 300 MHz Bruker spectrometer at ambient temperature. Solutions were typically prepared in either deuterochloroform (CDCl_3_) or deuterated dimethylsulfoxide (DMSO-*d*_6_) with chemical shifts referenced to deuterated solvent as an internal standard. ^1^H NMR data are reported indicating the chemical shift (*δ*), the multiplicity (s, singlet; d, doublet; t, triplet; q, quartet; m, multiplet; br, broad; dd, doublet of doublets, etc.), the coupling constant (*J*) in Hz and the integration (e.g. 1H). Deuterated solvents were obtained from the Sigma-Aldrich Chemical Co., Goss or Fluorochem. LC−MS spectra with UV detection were recorded on a Waters Acquity UPLC. Mass spectrometry was performed on a Waters Acquity SQD quadrupole spectrometer running in dual ES+ and ES− mode. High pH runs were conducted at pH 10 and low pH runs were conducted at pH 4, with a run time of 2 min. The column temperature was 40 °C, and the flow rate was 0.6 mL/min. Further details, including solvent gradients, are given in the Supporting Information. Details of the preparative HPLC instrument and the solvent gradient used to purify compounds are also given in the Supporting Information. All compounds were ≥95% purity as determined by examination of both the LC–MS and ^1^H NMR spectra unless otherwise indicated. When Cl or Br was present, expected isotopic distribution patterns were observed.

#### 5.1.1 General procedure for synthesis of quinazolines

4-Chloro-6,7-dimethoxyquinazoline **3** and the required nucleophile were heated in solvent either thermally or using microwave heating until no further reaction was observed. On cooling, the hydrochloride salt was isolated by filtration. Alternative isolation procedures were employed if precipitation did not occur. Additional purification by preparative HPLC or flash column chromatography was employed in some cases. Spectroscopic data for compounds **4** [13], **6**–**9** [14], [15], [16], **20**–**21** [13], **25** [13], **28** [17] and **30** [18] are in agreement with those reported in the literature.

##### 5.1.1.1 2-((6,7-Dimethoxyquinazolin-4-yl)amino)phenol hydrochloride (**5**)

A mixture of **3** (100 mg, 0.45 mmol) and 2-aminophenol (49 mg, 0.45 mmol) in MeCN afforded **5** (90 mg, 55%) as a yellow solid. ^1^H NMR (300 MHz, DMSO-*d*_6_) *δ* 11.09 (br s, 1H), 9.90 (br s, 1H), 8.72 (s, 1H), 8.22 (s, 1H), 7.36 (s, 1H), 7.32 (dd, *J* = 1.60, 7.82 Hz, 1H), 7.20–7.28 (m, 1H), 7.04 (dd, *J* = 1.18, 8.15 Hz, 1H), 6.91 (dt, *J* = 1.27, 7.56 Hz, 1H), 4.00 (s, 3H), 3.99 (s, 3H).

##### 5.1.1.2 3-((6,7-Dimethoxyquinazolin-4-yl)amino)benzene-1,2-diol hydrochloride (**10**)

A mixture of **3** (898 mg, 4.0 mmol) and 3-amino-1,2-benzenediol (500 mg, 4.0 mmol) in MeCN afforded **10** (1.24 g, 89%) as a dark brown solid. ^1^H NMR (300 MHz, DMSO-*d*_6_) *δ* 10.96 (br s, 1H), 9.54 (s, 1H), 8.99 (br s, 1H), 8.71 (s, 1H), 8.16 (s, 1H), 7.31 (s, 1H), 6.84 (dd, *J* = 2.17, 7.16 Hz, 1H), 6.69–6.81 (m, 2H), 4.00 (s, 3H), 3.98 (s, 3H). ^13^C NMR (75 MHz, DMSO-*d*_6_): *δ* 158.9, 156.0, 149.9, 148.5, 146.5, 141.0, 135.4, 124.4, 118.5, 118.2, 114.6, 106.9, 103.9, 99.9, 563, 56.3. HRMS (ESI) *m/z* [M + H]^+^ calcd for C_16_H_15_N_3_O_4_: 314.1140. Found: 314.1141.

##### 5.1.1.3 2-Bromo-3-((6,7-dimethoxyquinazolin-4-yl)amino)phenol hydrochloride (**11**)

A mixture of **3** (200 mg, 0.89 mmol), 3-amino-2-bromo-phenol [19] (167 mg, 0.89 mmol) and 5–6N HCl in IPA (0.01 mL) in IPA afforded **11** (310 mg, 92%) as a cream solid. ^1^H NMR (300 MHz, DMSO-*d*_6_) *δ* 11.30 (br s, 1H), 10.62 (s, 1H), 8.75 (s, 1H), 8.14 (s, 1H), 7.29–7.35 (m, 2H), 7.04 (dd, *J* = 1.41, 8.19 Hz, 1H), 6.97 (dd, *J* = 1.41, 7.82 Hz, 1H), 4.01 (s, 3H), 3.99 (s, 3H). ^13^C NMR (75 MHz, DMSO-*d*_6_): *δ* 159.1, 156.4, 155.5, 150.2, 148.6, 136.8, 135.3, 128.3, 119.6, 115.4, 110.0, 106.6, 103.6, 99.7, 56.7, 56.5. HRMS (ESI) *m/z* [M + H]^+^ calcd for C_16_H_14_BrN_3_O_3_: 376.0297. Found: 376.0297.

##### 5.1.1.4 2-Chloro-3-((6,7-dimethoxyquinazolin-4-yl)amino)phenol hydrochloride (**12**)

A mixture of **3** (1.56 g, 6.97 mmol) and 3-amino-2-chlorophenol (1.0 g, 6.97 mmol) in MeCN afforded **12** (2.21 g, 86%) as a beige solid. ^1^H NMR (300 MHz, DMSO-*d*_6_) *δ* 11.45 (br s, 1H), 10.59 (s, 1H), 8.77 (s, 1H), 8.22 (s, 1H), 7.36 (s, 1H), 7.27 (t, *J* = 7.72 Hz, 1H), 7.08 (dd, *J* = 1.22, 8.29 Hz, 1H), 6.98 (dd, *J* = 1.13, 7.82 Hz, 1H), 4.01 (s, 3H), 4.00 (s, 3H). ^13^C NMR (75 MHz, DMSO-*d*_6_): *δ* 157.5, 154.1, 153.1, 148.7, 146.7, 137.4, 126.8, 119.5, 118.2, 113.9, 108.5, 107.0, 101.9, 56.0, 55.7. HRMS (ESI) *m/z* [M + H]^+^ calcd for C_16_H_14_ClN_3_O_3_: 332.0802. Found: 332.0805.

##### 5.1.1.5 3-((6,7-Dimethoxyquinazolin-4-yl)amino)-2-methylphenol hydrochloride (**13**)

A mixture of **3** (500 mg, 2.23 mmol) and 3-amino-2-methylphenol (274 mg, 2.23 mmol) in IPA afforded **13** (708 mg, 91%) as a purple solid. ^1^H NMR (300 MHz, DMSO-*d*_6_) *δ* 11.37 (s, 1H), 9.73 (s, 1H), 8.70 (s, 1H), 8.27 (s, 1H), 7.36 (s, 1H), 7.11 (t, *J* = 7.96 Hz, 1H), 6.90 (d, *J* = 8.01 Hz, 1H), 6.77 (d, *J* = 7.72 Hz, 1H), 3.99 (s, 6H), 1.98 (s, 3H). ^13^C NMR (75 MHz, DMSO-*d*_6_): *δ* 158.9, 156.2 ( × 2), 150.1, 148.7, 136.2, 134.9, 126.2, 121.7, 117.9, 114.0, 106.5, 103.9, 99.5, 56.7, 56.4. HRMS (ESI) *m/z* [M + H]^+^ calcd for C_17_H_17_N_3_O_3_: 312.1348. Found: 312.1362.

##### 5.1.1.6 3-((6,7-Dimethoxyquinazolin-4-yl)amino)-2-fluorophenol (**14**)

A mixture of **3** (75 mg, 0.33 mmol) and 3-amino-2-fluorophenol (42 mg, 0.33 mmol) in MeCN returned the crude product which was purified by preparative HPLC (high pH) to afford **14** (21 mg, 18%) as a white solid. ^1^H NMR (300 MHz, DMSO-*d*_6_) *δ* 9.90 (s, 1H), 9.54 (br s, 1H), 8.36 (s, 1H), 7.83 (s, 1H), 7.18 (s, 1H), 6.97–7.05 (m, 1H), 6.84–6.93 (m, 2H), 3.94 (s, 6H).

##### 5.1.1.7 3-((6,7-Dimethoxyquinazolin-4-yl)amino)-2-methoxyphenol (**15**)

A suspension of **3** (20 mg, 0.09 mmol), **46h** (14 mg, 0.10 mmol) and 5–6N HCl in IPA (0.02 mL) in IPA was heated at 100 °C for 30 min in the microwave. The reaction was concentrated to dryness then partitioned between EtOAc and NaHCO_3_. A precipitate formed which was collected by filtration, washed with water (5 mL) and ether (5 mL) and dried under vacuum to give **15** (7 mg, 23%) as a white solid. ^1^H NMR (300 MHz, DMSO-*d*_6_) *δ* 9.38 (s, 1H), 9.18 (s, 1H), 8.32 (s, 1H), 7.81 (s, 1H), 7.16 (s, 1H), 6.90–6.99 (m, 2H), 6.77 (dd, *J* = 2.26, 7.44 Hz, 1H), 3.93 (s, 3H), 3.93 (s, 3H), 3.65 (s, 3H).

##### 5.1.1.8 3-((6,7-Dimethoxyquinazolin-4-yl)amino)-2-ethylphenol (**16**)

A mixture of **3** (115 mg, 0.51 mmol), **46a** (70 mg, 0.51 mmol) and 5–6N HCl in IPA (0.03 mL) in IPA returned the crude product which was purified by preparative HPLC to afford **16** (1.6 mg, 1%) as an off white solid. ^1^H NMR (300 MHz, DMSO-*d*_6_) *δ* 9.41 (br s, 1H), 9.27 (s, 1H), 8.23 (s, 1H), 7.83 (s, 1H), 7.14 (s, 1H), 7.04 (t, *J* = 7.97 Hz, 1H), 6.78 (d, *J* = 7.77 Hz, 1H), 6.70 (d, *J* = 7.68 Hz, 1H), 3.92 (s, 3H), 3.91 (s, 3H), 0.97 (t, *J* = 7.44 Hz, 3H) – CH_2_ of Et obscured under DMSO solvent peak.

##### 5.1.1.9 3-((6,7-Dimethoxyquinazolin-4-yl)amino)-2-(trifluoromethyl)phenol (**17**)

A mixture of **3** (52 mg, 0.23 mmol), **51** (61 mg, 0.23 mmol) and 5–6N HCl in IPA (0.03 mL) in IPA returned the crude product which was purified by preparative HPLC to afford *N*-[3-benzyloxy-2-(trifluoromethyl)phenyl]-6,7-dimethoxy-quinazolin-4-amine (32 mg, 31%) as a white solid. ^1^H NMR (300 MHz, DMSO-*d*_6_) *δ* 9.57 (s, 1H), 8.27 (s, 1H), 7.79 (s, 1H), 7.63 (t, *J* = 8.44 Hz, 1H), 7.29–7.51 (m, 6H), 7.17 (s, 1H), 7.04 (d, *J* = 8.19 Hz, 1H), 5.31 (s, 2H), 3.93 (s, 6H). A solution of *N*-[3-benzyloxy-2-(trifluoromethyl)phenyl]-6,7-dimethoxy-quinazolin-4-amine (27 mg, 0.06 mmol) in TFA (0.5 mL, 6.53 mmol) was heated for 45 min at 75 °C in the microwave. The yellow solution produced was concentrated under reduced pressure and purified by preparative HPLC to afford **17** (9 mg, 42%) as a yellow solid. ^1^H NMR (300 MHz, DMSO-*d*_6_) *δ* 10.53 (br s, 1H), 9.47 (s, 1H), 8.25 (s, 1H), 7.78 (s, 1H), 7.45 (t, *J* = 8.15 Hz, 1H), 7.16 (s, 1H), 6.99 (d, *J* = 8.19 Hz, 1H), 6.85 (d, *J* = 7.91 Hz, 1H), 3.92 (s, 3H), 3.92 (s, 3H).

##### 5.1.1.10 2-((6,7-Dimethoxyquinazolin-4-yl)amino)-6-hydroxybenzonitrile (**18**)

A mixture of **3** (126 mg, 0.56 mmol), **46e** (75 mg, 0.56 mmol) and 5–6N HCl in IPA (0.01 mL) in IPA returned the crude product. The crude material was dissolved in 7N ammonia in methanol, preabsorbed onto silica and purified by flash column chromatography eluting with 0–100% ethyl acetate in isohexane then 0–10% methanol in ethyl acetate to return **18** (11 mg, 6%) as a pale yellow solid. ^1^H NMR (300 MHz, DMSO-*d*_6_) *δ* 13.07 (d, *J* = 6.22 Hz, 1H), 9.47 (s, 1H), 9.08 (d, *J* = 5.93 Hz, 1H), 7.99 (s, 1H), 7.37–7.44 (m, 2H), 7.12 (d, *J* = 8.01 Hz, 1H), 6.51 (d, *J* = 8.67 Hz, 1H), 4.02 (s, 3H), 3.98 (s, 3H).

##### 5.1.1.11 5-((6,7-Dimethoxyquinazolin-4-yl)amino)-2-fluorophenol hydrochloride (**19**)

A mixture of **3** (75 mg, 0.33 mmol) and 5-amino-2-fluorophenol (42 mg, 0.33 mmol) in MeCN afforded **19** (75 mg, 64%) as an off-white solid. ^1^H NMR (300 MHz, DMSO-*d*_6_) *δ* 11.27 (s, 1H), 10.24 (br s, 1H), 8.81 (s, 1H), 8.27 (s, 1H), 7.35 (s, 1H), 7.32 (dd, *J* = 2.54, 8.01 Hz, 1H), 7.24 (dd, *J* = 8.76, 11.02 Hz, 1H), 7.05–7.11 (m, 1H), 4.01 (s, 3H), 3.99 (s, 3H).

##### 5.1.1.12 5-((6,7-Dimethoxyquinazolin-4-yl)amino)-2-methoxyphenol hydrochloride (**22**)

A mixture of **3** (100 mg, 0.45 mmol) and 5-amino-2-methoxyphenol (62 mg, 0.45 mmol) in MeCN afforded **22** (141 mg, 87%) as a yellow solid. ^1^H NMR (300 MHz, DMSO-*d*_6_) *δ* 11.07 (s, 1H), 9.32 (br s, 1H), 8.78 (s, 1H), 8.18 (s, 1H), 7.30 (s, 1H), 7.10 (s, 1H), 6.98–7.07 (m, 2H), 4.00 (s, 6H), 3.82 (s, 3H).

##### 5.1.1.13 3-((6,7-Dimethoxyquinazolin-4-yl)amino)-5-fluorophenol hydrochloride (**23**)

A mixture of **3** (75 mg, 0.33 mmol) and **46f** (68 mg, 0.40 mmol) in MeCN afforded **23** (18 mg, 15%) as a beige solid. ^1^H NMR (300 MHz, DMSO-*d*_6_) *δ* 11.14 (br s, 1H), 10.25 (br s, 1H), 8.88 (s, 1H), 8.25 (s, 1H), 7.34 (s, 1H), 7.12 (td, *J* = 1.85, 10.62 Hz, 1H), 7.01–7.06 (m, 1H), 6.54 (td, *J* = 2.11, 10.76 Hz, 1H), 4.02 (s, 3H), 4.00 (s, 3H).

##### 5.1.1.14 3-((6,7-Dimethoxyquinazolin-4-yl)amino)-5-methylphenol hydrochloride (**24**)

A mixture of **3** (75 mg, 0.33 mmol) and 3-amino-5-methylphenol (41 mg, 0.33 mmol) in MeCN afforded **24** (76 mg, 66%) as a green solid. ^1^H NMR (300 MHz, DMSO-*d*_6_) *δ* 11.19 (s, 1H), 9.65 (br s, 1H), 8.81 (s, 1H), 8.28 (s, 1H), 7.38 (s, 1H), 6.95 (s, 1H), 6.90 (s, 1H), 6.57 (s, 1H), 4.01 (s, 3H), 3.99 (s, 3H), 2.27 (s, 3H).

##### 5.1.1.15 4-Chloro-3-((6,7-dimethoxyquinazolin-4-yl)amino)phenol (**26**)

A mixture of **3** (75 mg, 0.33 mmol) and 3-amino-4-chlorophenol (48 mg, 0.33 mmol) in MeCN returned the crude product which was purified by preparative HPLC (high pH) to afford **26** (8 mg, 6%) as a white solid. ^1^H NMR (300 MHz, DMSO-*d*_6_) *δ* 9.82 (br s, 1H), 9.39 (s, 1H), 8.33 (s, 1H), 7.81 (s, 1H), 7.33 (d, *J* = 8.67 Hz, 1H), 7.18 (s, 1H), 6.96 (d, *J* = 2.83 Hz, 1H), 6.73 (dd, *J* = 2.83, 8.67 Hz, 1H), 3.93 (s, 6H).

##### 5.1.1.16 3-((6,7-Dimethoxyquinazolin-4-yl)amino)-4-methylphenol hydrochloride (**27**)

A mixture of **3** (100 mg, 0.45 mmol) and 2-amino-4-hydroxytoluene (60 mg, 0.49 mmol) in MeCN afforded **27** (81 mg, 48%) as a pale brown solid. ^1^H NMR (300 MHz, DMSO-*d*_6_) *δ* 11.30 (s, 1H), 9.57 (br s, 1H), 8.72 (s, 1H), 8.27 (s, 1H), 7.36 (s, 1H), 7.16 (d, *J* = 8.52 Hz, 1H), 6.72–6.78 (m, 2H), 4.00 (s, 6H), 2.07 (s, 3H).

##### 5.1.1.17 2,4-Dichloro-5-((6,7-dimethoxyquinazolin-4-yl)amino)phenol hydrochloride (**29**)

A mixture of **3** (100 mg, 0.45 mmol) and 5-amino-2,4-dichlorophenol (79 mg, 0.45 mmol) in MeCN returned the crude product which was recrystallized from MeCN/MeOH to afford **29** (31 mg, 36%) as a white solid. ^1^H NMR (300 MHz, DMSO-*d*_6_) *δ* 11.36 (br s, 1H), 10.96 (s, 1H), 8.79 (s, 1H), 8.16 (s, 1H), 7.71 (s, 1H), 7.32 (s, 1H), 7.16 (s, 1H), 4.01 (s, 3H), 4.00 (s, 3H).

##### 5.1.1.18 4-Chloro-5-((6,7-dimethoxyquinazolin-4-yl)amino)-2-methylphenol hydrochloride (**31**)

A mixture of **3** (142 mg, 0.63 mmol) and 5-amino-4-chloro-2-methyl-phenol (100 mg, 0.63 mmol) in MeCN returned the crude product which was recrystallized from DCM/MeOH to afford **31** (215 mg, 89%) as an off-white solid. ^1^H NMR (300 MHz, DMSO-*d*_6_) *δ* 11.17 (br s, 1H), 9.99 (br s, 1H), 8.76 (s, 1H), 8.11 (br s, 1H), 7.25–7.36 (m, 2H), 6.91 (s, 1H), 4.01 (s, 3H), 3.99 (s, 3H), 2.18 (s, 3H).

##### 5.1.1.19 3-((6,7-Dimethoxyquinazolin-4-yl)amino)-2,6-difluorophenol hydrochloride (**32**)

A mixture of **3** (36 mg, 0.16 mmol) and **46b** (23 mg, 0.16 mmol) in MeCN afforded **32** (30 mg, 51%) as an off-white. ^1^H NMR (300 MHz, DMSO-*d*_6_) *δ* 11.30 (br s, 1H), 10.53 (br s, 1H), 8.80 (s, 1H), 8.19 (s, 1H), 7.33 (s, 1H), 7.14–7.22 (m, 1H), 6.93–7.02 (m, *J* = 5.51, 7.96 Hz, 1H), 4.01 (s, 4H), 4.00 (s, 3H).

##### 5.1.1.20 3-((6,7-Dimethoxyquinazolin-4-yl)amino)-2,4-difluorophenol (**33**)

A mixture of **3** (50 mg, 0.22 mmol), **46c** (39 mg, 0.27 mmol) and 4 N HCl in dioxane (0.2 mL, 0.80 mmol) in 1,4-dioxane was heated in the microwave at 100 °C for 30 min. The reaction mixture was cooled and evaporated to dryness. The residue was partitioned between DCM and sat. NaHCO_3_, separated and evaporated to dryness. The residue was purified by preparative HPLC to afford **33** (17 mg, 22%) as a white solid. ^1^H NMR (300 MHz, DMSO-*d*_6_) *δ* 9.93 (br s, 1H), 9.43 (s, 1H), 8.34 (s, 1H), 7.84 (s, 1H), 7.21 (s, 1H), 6.87–7.06 (m, 2H), 3.94 (s, 6H). HRMS (ESI) *m/z* [M + H]^+^ calcd for C_16_H_13_F_2_N_3_O_3_: 334.1003. Found: 334.1004.

##### 5.1.1.21 4-Chloro-3-((6,7-dimethoxyquinazolin-4-yl)amino)-2-fluorophenol (**34**)

A mixture of **3** (50 mg, 0.22 mmol), **46d** (43 mg, 0.27 mmol) and 4 N HCl in dioxane (0.2 mL, 0.80 mmol) in 1,4-dioxane was heated in the microwave at 100 °C for 30 min. The reaction was cooled and evaporated to dryness. The residue was partitioned between DCM and sat. NaHCO_3_, separated and evaporated to dryness. The residue was purified by preparative HPLC to afford **34** (7 mg, 9%) as a white solid. ^1^H NMR (300 MHz, DMSO-*d*_6_) *δ* 10.26 (br s, 1H), 9.47 (s, 1H), 8.31 (s, 1H), 7.86 (s, 1H), 7.23 (dd, *J* = 1.88, 8.95 Hz, 1H), 7.20 (s, 1H), 6.97 (t, *J* = 8.85 Hz, 1H), 3.94 (s, 6H). HRMS (ESI) *m/z* [M + H]^+^ calcd for C_16_H_13_ClFN_3_O_3_: 350.0708. Found: 350.0708.

##### 5.1.1.22 3-((6,7-Dimethoxyquinazolin-4-yl)amino)-5-fluoro-2-methylphenol hydrochloride (**35**)

A mixture of **3** (60 mg, 0.27 mmol), **46i** (40 mg, 0.28 mmol) and HCl in IPA (0.01 mL) in IPA afforded **35** (56 mg, 57%) as a white solid. ^1^H NMR (300 MHz, DMSO-*d*_6_) *δ* 11.33 (br s, 1H), 10.26 (s, 1H), 8.74 (s, 1H), 8.24 (s, 1H), 7.34 (s, 1H), 6.67–6.75 (m, 2H), 4.00 (s, 3H), 4.00 (s, 3H), 1.95 (s, 3H).

##### 5.1.1.23 3-((6,7-Dimethoxyquinazolin-4-yl)amino)-4-fluoro-2-methylphenol hydrochloride (**36**)

A mixture of **3** (80 mg, 0.35 mmol) and 3-amino-4-fluoro-2-methyl-phenol [20] (100 mg, 0.71 mmol) in MeCN afforded **36** (42 mg, 15%) as an off-white solid. ^1^H NMR (300 MHz, DMSO-*d*_6_) *δ* 11.12 (br s, 1H), 9.69 (s, 1H), 8.75 (s, 1H), 8.23 (s, 1H), 7.32 (s, 1H), 7.04 (t, *J* = 9.07 Hz, 1H), 6.88 (dd, *J* = 4.62, 8.95 Hz, 1H), 4.01 (s, 3H), 4.00 (s, 3H), 2.03 (s, 3H). ^13^C NMR (75 MHz, DMSO-*d*_6_): *δ* 159.4, 156.4, 152.5, 151.9 (×2), 149.4, 148.9, 135.4, 123.7 (×2), 123.5, 114.2, 114.1, 112.7, 112.4, 106.5, 103.8, 99.7, 56.8, 56.4. HRMS (ESI) *m/z* [M + H]^+^ calcd for C_17_H_16_FN_3_O_3_: 330.1254. Found: 330.1254.

##### 5.1.1.24 4-Chloro-3-((6,7-dimethoxyquinazolin-4-yl)amino)-2-methylphenol (**37**)

A mixture of **3** (50 mg, 0.22 mmol), 3-amino-4-chloro-2-methyl-phenol [21] (42 mg, 0.27 mmol) and 4N HCl in dioxane (0.2 mL, 0.8 mmol) in 1,4-dioxane was heated in the microwave at 100 °C for 30 min. The reaction was cooled and evaporated to dryness. The residue was partitioned between DCM and sat. NaHCO_3_, separated and evaporated to dryness. The residue was purified by preparative HPLC (high pH) to afford **37** (15 mg, 20%) as a white solid. ^1^H NMR (300 MHz, DMSO-*d*_6_) *δ* 9.73 (s, 1H), 9.37 (s, 1H), 8.24 (s, 1H), 7.86 (s, 1H), 7.21 (d, *J* = 8.51 Hz, 1H), 7.17 (s, 1H), 6.84 (d, *J* = 8.67 Hz, 1H), 3.93 (s, 6H), 1.99 (s, 3H).

##### 5.1.1.25 2-Chloro-3-((6,7-dimethoxyquinazolin-4-yl)amino)-6-methylphenol hydrochloride (**38**)

A mixture of **3** (500 mg, 2.23 mmol) and 3-amino-2-chloro-6-methylphenol (351 mg, 2.23 mmol) in IPA afforded **38** (713 mg, 84%) as a beige solid. ^1^H NMR (300 MHz, DMSO-*d*_6_) *δ* 11.52 (br s, 1H), 9.45 (s, 1H), 8.75 (s, 1H), 8.27 (s, 1H), 7.40 (s, 1H), 7.19 (d, *J* = 8.10 Hz, 1H), 6.93 (d, *J* = 8.01 Hz, 1H), 4.00 (s, 6H), 2.28 (s, 3H).

##### 5.1.1.26 2,4-Dichloro-3-((6,7-dimethoxyquinazolin-4-yl)amino)phenol (**39**)

A mixture of **3** (100 mg, 0.45 mmol), **46g** (158 mg, 0.90 mmol) and 4N HCl in dioxane (0.2 mL, 0.80 mmol) in 1,4-dioxane was heated in the microwave at 140 °C for 5 h. The reaction was cooled and evaporated to dryness. The residue was partitioned between DCM/IPA and sat. NaHCO_3_, separated and evaporated to dryness. The residue was purified by flash chromatography eluting with an isohexane/ethyl acetate gradient to afford **39** (8 mg, 5%) as a pale yellow solid. ^1^H NMR (300 MHz, DMSO-*d*_6_) *δ* 10.56 (s, 1H), 9.58 (s, 1H), 8.27 (s, 1H), 7.86 (s, 1H), 7.38 (d, *J* = 8.85 Hz, 1H), 7.19 (s, 1H), 7.01 (d, *J* = 8.85 Hz, 1H), 3.94 (s, 6H).

##### 5.1.1.27 2-Chloro-3-((6,7-dimethoxyquinazolin-4-yl)amino)-4-fluorophenol (**40**)

A mixture of **3** (100 mg, 0.45 mmol), 3-amino-2-chloro-4-fluoro-phenol [20] (108 mg, 0.67 mmol) and 4N HCl in dioxane (0.4 mL, 1.6 mmol) in 1,4-dioxane was heated in the microwave at 100 °C for 90 min. The reaction was cooled, diluted with DCM and washed with sat. NaHCO_3_, separated and evaporated to dryness. The residue was purified by preparative HPLC to afford **40** (26 mg, 17%) as a white solid. ^1^H NMR (300 MHz, DMSO-*d*_6_) *δ* 10.22 (s, 1H), 9.46 (s, 1H), 8.29 (s, 1H), 7.86 (s, 1H), 7.13–7.22 (m, 2H), 6.96 (dd, *J* = 4.85, 9.09 Hz, 1H), 3.94 (s, 6H). ^13^C NMR (75 MHz, DMSO-*d*_6_): *δ* 157.4, 154.3, 153.1, 150.3, 147.8, 146.8, 125.5, 125.3, 119.9, 114.1, 113.8, 108.3, 107.0, 101.8, 56.0, 55.8. HRMS (ESI) *m/z* [M + H]^+^ calcd for C_16_H_13_ClFN_3_O_3_: 350.0708. Found: 350.0708.

##### 5.1.1.28 3-((6,7-Dimethoxyquinazolin-4-yl)(methyl)amino)phenol hydrochloride (**41**)

A mixture of **3** (84 mg, 0.37 mmol) and **53** (140 mg, 0.97 mmol) in MeCN afforded **41** (88 mg, 68%) as a light brown solid. ^1^H NMR (300 MHz, DMSO-*d*_6_) *δ* 10.17 (br s, 1H), 8.97 (s, 1H), 7.36–7.43 (m, 2H), 6.90–6.99 (m, 3H), 6.34 (s, 1H), 3.92 (s, 3H), 3.70 (s, 3H), 3.26 (s, 3H).

##### 5.1.1.29 3-((6,7-Dimethoxyquinazolin-4-yl)oxy)phenol (**42**)

A mixture of **3** (200 mg, 0.89 mmol), resorcinol (118 mg, 1.07 mmol) and potassium carbonate (390 mg, 2.82 mmol) in MeCN (10 mL) was heated at 120 °C in the microwave for 30 min. The reaction mixture was concentrated to dryness, suspended in water then extracted with DCM. The organic phase was concentrated to dryness then purified by preparative HPLC to afford **42** (52 mg, 20%) as a white solid. ^1^H NMR (300 MHz, DMSO-*d*_6_) *δ* 9.80 (br s, 1H), 8.56 (s, 1H), 7.53 (s, 1H), 7.38 (s, 1H), 7.26 (t, *J* = 8.05 Hz, 1H), 6.65–6.74 (m, 3H), 3.99 (s, 3H), 3.97 (s, 3H).

##### 5.1.1.30 3-((6,7-Dimethoxyquinazolin-4-yl)thio)phenol hydrochloride (**43**)

A mixture of **3** (100 mg, 0.45 mmol) and 3-mercaptophenol (0.05 mL, 0.45 mmol) in MeCN returned the crude product which was recrystallized from MeCN/MeOH to afford **43** (53 mg, 34%) as a white solid. ^1^H NMR (300 MHz, DMSO-*d*_6_) *δ* 9.72 (br. s, 1H), 8.74 (s, 1H), 7.28–7.38 (m, 3H), 7.01–7.07 (m, 2H), 6.92 (d, *J* = 8.14 Hz, 1H), 3.99 (s, 3H), 3.99 (s, 3H).

##### 5.1.1.31 1-(6,7-Dimethoxyquinazolin-4-yl)indolin-4-ol hydrochloride (**44**)

A mixture of **3** (250 mg, 1.11 mmol) and indolin-4-ol (150 mg, 1.11 mmol) in MeCN afforded **44** (350 mg, 88%) as a yellow solid. ^1^H NMR (300 MHz, DMSO-*d*_6_) *δ* 9.82 (br s,1H), 8.86 (s, 1H), 7.57 (s, 1H), 7.40 (s, 1H), 7.32 (d, *J* = 8.10 Hz, 1H), 7.11 (t, *J* = 8.10 Hz, 1H), 6.66 (d, *J* = 7.63 Hz, 1H), 4.71 (t, *J* = 7.54 Hz, 2H), 4.01 (s, 3H), 3.91 (s, 3H), 3.11 (t, *J* = 7.54 Hz, 2H).

##### 5.1.1.32 1-(6,7-Dimethoxyquinazolin-4-yl)-1,2,3,4-tetrahydroquinolin-5-ol hydrochloride (**45**)

A mixture of **3** (250 mg, 1.11 mmol), 1,2,3,4-tetrahydro-quinolin-5-ol hydrochloride (206 mg, 1.11 mmol) in MeCN (12 mL) afforded **45** (390 mg, 94%) as an orange solid. ^1^H NMR (300 MHz, DMSO-*d*_6_) *δ* 9.81 (br s, 1H), 8.93 (s, 1H), 7.31 (s, 1H), 6.88 (d, *J* = 8.10 Hz, 1H), 6.62–6.75 (m, 2H), 6.20–6.42 (m, 1H), 4.12 (d, *J* = 5.46 Hz, 2H), 3.96 (s, 3H), 3.43 (s, 3H), 2.79 (t, *J* = 6.64 Hz, 2H), 1.98–2.16 (m, 2H).

#### 5.1.2 Procedures for synthesis of anilines **46a**–**i**, **49h**, **49i**, **51** and **53**

##### 5.1.2.1 3-Amino-2-ethylphenol (**46a**)

To an ice-cooled solution of 2-ethyl-3-methoxyaniline [22] (141 mg, 0.93 mmol) in DCM (1 mL) was added BBr_3_ (1 M solution in DCM, 4.66 mL, 4.66 mmol) dropwise under nitrogen. The reaction mixture was stirred at room temperature overnight then poured into ice water, neutralized with sodium carbonate and separated. The aqueous phase was further extracted with DCM (2 × 20 mL) and the combined organics were dried over MgSO_4_, filtered and concentrated under reduced pressure to return **46a** (128 mg, 100%) as a brown solid which was used without further purification. ^1^H NMR (300 MHz, DMSO-*d*_6_) *δ* 8.75 (s, 1H), 6.64 (t, *J* = 7.9 Hz, 1H), 5.97–6.19 (m, 2H), 4.96 (br. s, 2H), 2.44 (q, *J* = 7.6 Hz, 2H), 0.98 (t, *J* = 7.6 Hz, 3H).

##### 5.1.2.2 3-Amino-2,6-difluorophenol (**46b**)

A mixture of 2,4-difluoro-3-methoxyaniline (500 mg, 3.14 mmol) and pyridinium chloride (1.0 g, 8.89 mmol) was heated at 200 °C for 1 h then allowed to cool to room temperature. The reaction mixture was diluted with water (50 mL) and extracted with DCM (50 mL). The organic phase was concentrated under reduced pressure under reduced pressure to return the crude product which was purified by flash column chromatography, eluting with 0–100% EtOAc in isohexane, then 1% 7N ammonia in methanol/EtOAc to return **46b** (23 mg, 5%). ^1^H NMR (300 MHz, DMSO-*d*_6_) *δ* 9.65 (s, 1H), 6.67 (ddd, *J* = 2.17, 8.90, 10.78 Hz, 1H), 6.15 (dt, *J* = 5.09, 9.18 Hz, 1H), 4.83 (s, 2H).

##### 5.1.2.3 3-Amino-2,4-difluorophenol (**46c**)

A mixture of 2,6-difluoro-3-methoxyaniline (1.0 g, 6.28 mmol) and pyridinium chloride (2.1 g, 17.79 mmol) was heated at 200 °C for 1 h then allowed to cool to room temperature. The reaction mixture was diluted with water (25 mL) and then neutralized with Na_2_HCO_3_. The resultant precipitate was isolated by filtration, washed with water and dried overnight at 40 °C under vacuum to return **46c** (470 mg, 52%) as a red solid. ^1^H NMR (300 MHz, DMSO-*d*_6_) *δ* 9.32 (s, 1H), 6.64 (ddd, *J* = 2.17, 8.92, 10.86 Hz, 1H), 6.07 (dt, *J* = 5.04, 9.16 Hz, 1H), 5.03 (s, 2H).

##### 5.1.2.4 3-Amino-4-chloro-2-fluorophenol (**46d**)

A mixture of 6-chloro-2-fluoro-3-methoxyaniline (1.0 g, 5.7 mmol) and pyridinium chloride (1.97 g, 17.1 mmol) was heated at 200 °C for 1 h then allowed to cool to room temperature. The reaction mixture was diluted with water (25 mL) and then neutralized with sodium bicarbonate. The resultant precipitate was isolated by filtration, washed with water and dried overnight at 40 °C under vacuum to return **46d** (244 mg, 27%). ^1^H NMR (300 MHz, DMSO-*d*_6_) *δ* 9.64 (s, 1H), 6.82 (dd, *J* = 2.07, 8.85 Hz, 1H), 6.17 (t, *J* = 8.81 Hz, 1H), 5.17 (s, 2H).

##### 5.1.2.5 2-Amino-6-hydroxybenzonitrile (**46e**)

A solution of 2-hydroxy-6-nitrobenzonitrile [23] (1.3 g, 7.92 mmol) in ethanol (100 mL) was treated with 5% Pd/C (450 mg). The flask was evacuated and filled with nitrogen three times, then evacuated and filled with hydrogen three times. The reaction mixture was stirred under an atmosphere of hydrogen at room temperature overnight, then flushed with nitrogen and filtered through Celite. The filtrate was concentrated under reduced pressure to return **46e** (910 mg, 77%). ^1^H NMR (300 MHz, DMSO-*d*_6_) *δ* 10.39 (br. s., 1H), 7.03 (t, *J* = 8.19 Hz, 1H), 6.17 (d, *J* = 8.23 Hz, 1H), 6.06 (d, *J* = 8.20 Hz, 1H), 5.79 (s, 2H).

##### 5.1.2.6 3-Amino-5-fluorophenol (**46f**)

To a solution of 3-fluoro-5-nitrophenol (1.0 g, 6.37 mmol) in ethanol (20 mL) and acetic acid (20 mL) was added iron powder (0.36 g, 6.37 mmol) and the mixture heated at reflux under nitrogen for 2 h then cooled to room temperature and filtered through Celite. The filtrate was concentrated under reduced pressure to return the crude product which was purified by flash column chromatography, eluting with 0–20% MeOH in DCM to return **46f** (820 mg, 86%) as a brown oil which solidified on standing. ^1^H NMR (300 MHz, DMSO-*d*_6_) *δ* 5.82 (t, *J* = 1.93 Hz, 1H), 5.78 (dt, *J* = 11.60, 2.00 Hz, 1H), 5.69 (dt, *J* = 11.11, 2.07 Hz, 1H).

##### 5.1.2.7 3-Amino-2,4-dichlorophenol (**46g**)

To a stirred solution of 2,4-dichloro-3-nitrophenol (2.0 g, 9.62 mmol) in ethanol (50 mL) was added iron powder (2.15 g, 38.46 mmol), ammonium chloride (2.06 g, 38.46 mmol) and water (25 mL). The reaction heated at reflux for 6 h, cooled a little, then filtered through Celite and washed with hot ethanol. The filtrate was concentrated under reduced pressure to remove ethanol, then extracted with DCM (3 × 25 mL), dried over MgSO_4_, filtered and concentrated under reduced pressure to return the crude product which was purified by flash column chromatography, eluting with 0–20% EtOAc in isohexane to return **46g** (1.45 g, 85%) as a brown solid. ^1^H NMR (300 MHz, DMSO-*d*_6_) *δ* 10.01 (s, 1H), 6.99 (d, *J* = 8.67 Hz, 1H), 6.24 (d, *J* = 8.76 Hz, 1H), 5.28 (br s, 2H).

##### 5.1.2.8 3-Amino-2-methoxyphenol (**46h**)

To a solution of **49h** (358 mg, 1.44 mmol) in THF (10 mL) was added water (5 mL) and sodium perborate (569 mg, 3.7 mmol). The mixture was stirred at ambient temperature overnight. Excess THF was removed under reduced pressure. The residue was stirred with sat. NH_4_Cl solution (10 mL) and DCM (50 mL) for 1 h, then separated. The organics were concentrated under reduced pressure and purified by flash column chromatography, eluting with 0–60% EtOAc in isohexane, to return **46h** (120 mg, 57%) as an off-white solid. ^1^H NMR (300 MHz, DMSO-*d*_6_) *δ* 8.80 (s, 1H), 6.55 (t, *J* = 7.84 Hz, 1H), 6.12 (d, *J* = 7.95 Hz, 1H), 6.05 (d, *J* = 7.77 Hz, 1H), 4.71 (s, 2H), 3.62 (s, 3H).

##### 5.1.2.9 3-Amino-5-fluoro-2-methylphenol (**46i**)

To a solution of **49i** (761 mg, 1.8 mmol) in THF (40 mL) was added water (20 mL) and sodium perborate (961 mg, 6.2 mmol). The mixture was stirred at ambient temperature overnight. THF was removed under reduced pressure and the aqueous extracted with DCM (50 mL). The organics were concentrated under reduced pressure and purified by flash column chromatography, eluting with 0–30% EtOAc in isohexane, to return **46i** (225 mg, 85%) as a light brown solid. ^1^H NMR (300 MHz, DMSO-*d*_6_) *δ* 9.20 (s, 1H), 5.79–5.93 (m, 2H), 4.99 (s, 2H), 1.81 (s, 3H).

##### 5.1.2.10 2-Methoxy-3-(4,4,5,5-tetramethyl-1,3,2-dioxaborolan-2-yl)aniline (**49h**)

A suspension of 3-bromo-2-methoxyaniline (500 mg, 2.47 mmol), bis(pinacolato)diboron (755 mg, 2.97 mmol) and potassium acetate (729 mg, 7.42 mmol) in 1,4-dioxane (8 mL) was sparged with nitrogen for 5 min. Pd(dppf)Cl_2_·DCM (173 mg, 0.21 mmol) was added and the mixture was heated under reflux for 2.5 h then cooled to room temperature. The reaction was diluted with DCM (35 mL) and washed with water (35 mL). The organic phase was concentrated under reduced pressure then purified by flash column chromatography, eluting with 0–100% EtOAc in isohexane, to return **49h** (358 mg, 58%) an off-white solid. ^1^H NMR (300 MHz, DMSO-*d*_6_) *δ* 6.72–6.83 (m, 3H), 4.80 (s, 2H), 3.64 (s, 3H), 1.28 (s, 12H).

##### 5.1.2.11 5-Fluoro-2-methyl-3-(4,4,5,5-tetramethyl-1,3,2-dioxaborolan-2-yl)aniline (**49i**)

A suspension of 3-bromo-5-fluoro-2-methyl-aniline [24] (725 mg, 3.55 mmol), bis(pinacolato)diboron (1.1 g, 4.26 mmol) and potassium acetate (1.0 g, 10.66 mmol) in 1,4-dioxane (35 mL) was sparged with nitrogen for 5 min. Pd(dppf)Cl_2_·DCM (174 mg, 0.21 mmol) was added and the mixture was heated under reflux for 4 h. The reaction was cooled to room temperature and concentrated under reduced pressure. The residue was diluted with EtOAc, filtered through Celite, then concentrated under reduced pressure to return the crude product which was purified by flash column chromatography, eluting with 0–50% EtOAc in isohexane, to return **49i** (761 mg, 78%). ^1^H NMR (300 MHz, CDCl_3_) *δ* 6.90 (dd, *J* = 2.68, 9.18 Hz, 1H), 6.49 (dd, *J* = 2.64, 10.17 Hz, 1H), 2.33 (s, 3H), 1.36 (s, 12H).

##### 5.1.2.12 1-Benzyloxy-3-bromo-2-(trifluoromethyl)benzene (**50**)

To an ice-cooled solution of benzyl alcohol (2.19 mL, 21.2 mmol) in DMF (30 mL) was added sodium hydride (0.97 g 60% dispersion on mineral oil, 24.23 mmol) portionwise. The mixture was stirred for 1 h before adding 3-fluoro-2-(trifluoromethyl)bromobenzene (4.91 g, 20.19 mmol) portionwise. The mixture was stirred at ambient temperature overnight, then diluted with EtOAc (50 mL) and washed with water (3 × 50 mL) and brine (50 mL). The organics were concentrated under reduced pressure then purified by flash column chromatography, eluting with 0–30% EtOAc in isohexane, to return **50** (5.6 g, 84%) as a colourless oil. ^1^H NMR (300 MHz, CDCl_3_) *δ* 6.85–7.77 (m, 8H), 4.92–5.25 (m, 2H).

##### 5.1.2.13 3-(Benzyloxy)-2-(trifluoromethyl)aniline (**51**)

A mixture of **50** (5.6 g, 16.79 mmol), benzophenone imine (3.1 mL, 18.47 mmol), cesium carbonate (4.88 g, 25.19 mmol), Pd(OAc)_2_ (188 mg, 0.8400 mmol) and BINAP (1.05 g, 1.68 mmol) in toluene (65 mL) was flushed with nitrogen and heated under reflux overnight. The reaction mixture was cooled and filtered, then the filtrate was diluted with EtOAc (50 mL) and washed successively with 0.5 M HCl (2 × 25 mL), sat. NaHCO_3_ (2 × 25 mL) and brine (25 mL). The organics were dried over MgSO_4_, filtered and concentrated under reduced pressure to give the crude product which was purified by flash column chromatography, eluting with 0–30% EtOAc in isohexane, to return *N*-[3-benzyloxy-2-(trifluoromethyl)phenyl]-1,1-diphenyl-methanimine (4.7 g, 64%) as an orange solid. Analysis indicated the product contained some aromatic impurities and was progressed without additional purification. ^1^H NMR (300 MHz, CDCl_3_) *δ* 7.77 (br d, *J* = 7.44 Hz, 2H), 7.05–7.56 (m, 14H), 6.59 (d, *J* = 8.38 Hz, 1H), 6.19 (d, *J* = 8.01 Hz, 1H), 5.10 (s, 2H). To a solution of crude *N*-[3-benzyloxy-2-(trifluoromethyl)phenyl]-1,1-diphenyl-methanimine (600 mg, 1.39 mmol) in THF (15 mL) was added 2 M HCl (3 mL). The reaction mixture was stirred overnight at ambient temperature then diluted with EtOAc (50 mL) and washed with sat. NaHCO_3_ (2 × 25 mL). The organics were concentrated under reduced pressure, then purified by flash column chromatography, eluting with 0–25% EtOAc in isohexane, to return **51** (286 mg, 77%) as a yellow oil. ^1^H NMR (300 MHz, DMSO-*d*_6_) *δ* 7.28–7.47 (m, 5H), 7.13 (t, *J* = 8.24 Hz, 1H), 6.40 (d, *J* = 8.48 Hz, 1H), 6.35 (d, *J* = 8.10 Hz, 1H), 5.59 (s, 2H), 5.11 (s, 2H).

##### 5.1.2.14 3-(Methylamino)phenol (**53**)

To a solution of **52** [25] (1.07 g, 5.11 mmol) in THF (20 mL) was added LiAlH_4_ (1.3 g, 30.96 mmol) portionwise. The mixture was heated under reflux for 4 h, then cooled to ambient temperature. Sat. Na_2_SO_4_ (30 mL) was cautiously added and the reaction mixture stirred vigorously for 1 h before being acidified with 2 M HCl. The reaction mixture was extracted with EtOAc (50 mL), then washed with brine (25 mL) and dried over MgSO_4_, filtered and concentrated under reduced pressure to return **53** (140 mg, 22%). ^1^H NMR (300 MHz, DMSO-*d*_6_) *δ* 8.88 (br s, 1H), 6.73–6.88 (m, 1H), 5.90–6.01 (m, 3H), 5.45 (br s, 1H), 2.60 (s, 3H).

### 5.2 Biological evaluation

#### 5.2.1 Biochemical assay

Kinase activity was detected using CisBio HTRF kinEASE kit based on time-resolved fluorescence transfer (FRET). The assay was performed in 384-well white plates (Corning #3574) in a reaction volume of 10 μL containing 1× CisBio enzymatic buffer supplemented with a final concentration of 5 mM MgCl_2_, 1 mM DTT, 10 nM SEB and 0.01% Triton X100 for RET. The same buffer was used for the KDR biochemical assay with the addition of 2 mM MnCl_2_.

Inhibitors were pre-incubated in the plate for 15 min with 5 μL kinase and assay buffer at the following concentrations; 13 pM RET (Carna Biosciences; 08-159) and 150 pM KDR (Millipore; 14-630). The reaction was initiated by the addition of 5 μL ATP and substrate at 2× final reaction concentrations. For RET, this was 18 μM and 2 μM; for KDR, this was 16 μM and 1 μM, respectively. Reactions were performed at ATP K_m_ for each target. The assay was allowed to proceed at room temperature for 20 min before terminating with the addition of 10 μL HTRF detection buffer containing EDTA supplemented with TK-antibody labelled with Eu^3+^-Cryptate (1:100 dilution) and streptavidin-XL665 (128 nM). Following incubation at room temperature for 1 h, FRET signal was measured using the Pherastar FS Microplate Reader.

#### 5.2.2 BaF3 cellular assay

The system originally developed by Daley and Baltimore [26] was used, whereby IL3-dependent BaF3 cells are modified to express an activated recombinant kinase. Following removal of IL3, the modified cells are dependent on the activity of the recombinant kinase for survival and proliferation. The BaF3 cell lines, expressing KIF5B-RET (gift from Pasi Janne [27]) and KDR (Advanced Cellular Dynamics, San Diego) were maintained in RPMI-1640 media containing 10% FBS and appropriate antibiotics. Non-modified BaF3 cells (WT) were maintained in RPMI-1640 media containing 10% FBS and supplemented with 10 ng/mL recombinant mouse IL3 (R&D systems). For assessment of compound IC_50_, cells were plated into 384-well plates at 1500 or 3000 cells per well in 30 μL culture medium and compounds dispensed using an acoustic liquid handling platform (LABCYTE). Following incubation of the cells for 48 h at 37 °C in a humidified 5% CO_2_ atmosphere, viability was determined by addition of 10 μL CellTiter-Glo reagent (Promega) and measurement of luminescence.

#### 5.2.3 Pharmacokinetics

All studies were conducted after review by the Animal Welfare and Ethical Review Body at CRUK:MI and in accordance with the University of Manchester Policy on the use of animals in research. All work was carried out in compliance with the Animals (Scientific Procedures) Act 1986. Pharmacokinetics were studied in male CD-1 mice following single intravenous or oral administration. Blood samples were collected as dried blood spots and assayed following solvent extraction through a phospholipid removal plate followed by LC-MS/MS analysis. The resulting concentration-time data were analysed by non-compartmental methods (PK Solver, Excel Add-In).

## Figures and Tables

**Table 1:**
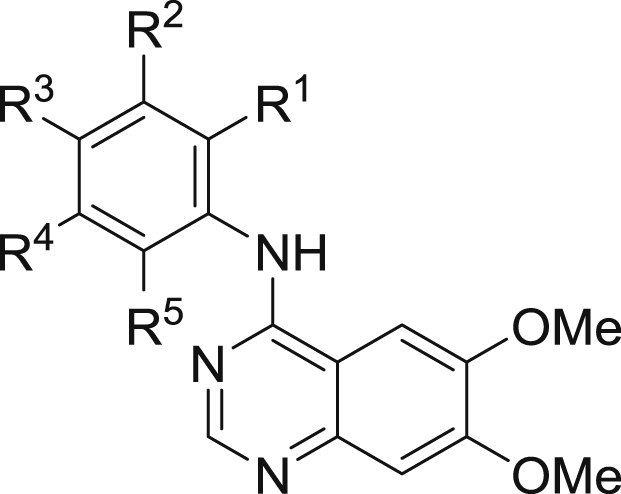
Biochemical data of selected anilinoquinazolines.^a^

Cpd	Ar					RET IC_50_ (nM)	KDR IC_50_ (nM)	Selectivity
R^1^	R^2^	R^3^	R^4^	R^5^
**1**	n/a					49 (29)	175 (86)	3
**2**	n/a					650 (580)	16 (31)	<1
**4**	F	H	Br	H	H	100 (16)	460 (44)	5
**5**	OH	H	H	H	H	1600 (220)	>10000	≥6
**6**	H	OH	H	H	H	4.5 (4.4)	66 (30)	15
**7**	H	H	OH	H	H	720 (48)	2800 (510)	4
**8**	H	NH_2_	H	H	H	590 (260)	7600 (2700)	13
**9**	H	OMe	H	H	H	1200 (330)	4800 (410)	4
**10**	OH	OH	H	H	H	3.9 (2)	2300 (1100)	590
**11**	Br	OH	H	H	H	23 (8.3)	4100 (2200)	178
**12**	Cl	OH	H	H	H	19 (5.9)	2500 (1600)	132
**13**	Me	OH	H	H	H	190 (76)	>10000	≥50
**14**	F	OH	H	H	H	10 (2.6)	280 (120)	28
**15**	OMe	OH	H	H	H	1100 (290)	>10000	≥9
**16**	Et	OH	H	H	H	3800 (18)	>10000	≥3
**17**	CF_3_	OH	H	H	H	5500 (2000)	>10000	≥2
**18**	CN	OH	H	H	H	>10000	>10000	nd
**19**	H	OH	F	H	H	3.2 (1.2)	72 (36)	23
**20**	H	OH	Me	H	H	4.6 (0.28)	18 (9.5)	4
**21**	H	OH	Cl	H	H	10 (8.6)	58 (14)	6
**22**	H	OH	OMe	H	H	11 (2.1)	73 (54)	7
**23**	H	OH	H	F	H	2.6 (1.1)	66 (71)	25
**24**	H	OH	H	Me	H	29 (22)	350 (300)	12
**25**	H	OH	H	H	F	1.1 (0.54)	20 (8.8)	18
**26**	H	OH	H	H	Cl	2.6 (0.78)	71 (45)	27
**27**	H	OH	H	H	Me	8.4 (1.9)	160 (53)	19
**28**	H	OH	F	H	F	0.41 (0.15)	9.5 (6.5)	23
**29**	H	OH	Cl	H	Cl	5 (2.8)	15 (4.3)	3
**30**	H	OH	Me	H	F	0.75 (0.071)	2.3 (0.071)	3
**31**	H	OH	Me	H	Cl	13 (4.4)	17 (8.8)	1
**32**	F	OH	F	H	H	2 (0.39)	230 (150)	115
**33**	F	OH	H	H	F	1.7 (0.33)	69 (14)	41
**34**	F	OH	H	H	Cl	4.8 (1.4)	230 (77)	48
**35**	Me	OH	H	F	H	21 (8.6)	5900 (1500)	281
**36**	Me	OH	H	H	F	44 (5.7)	5700 (1400)	130
**37**	Me	OH	H	H	Cl	120 (28)	>10000	≥83
**38**	Cl	OH	Me	H	H	9.3 (5.2)	360 (260)	39
**39**	Cl	OH	H	H	Cl	9.7 (13)	1400 (860)	144
**40**	Cl	OH	H	H	F	3.9 (0.5)	1100 (160)	282

a Biological values are expressed as the geometric mean of four independent determinations and standard deviations are quoted in parentheses.

**Table 2:**
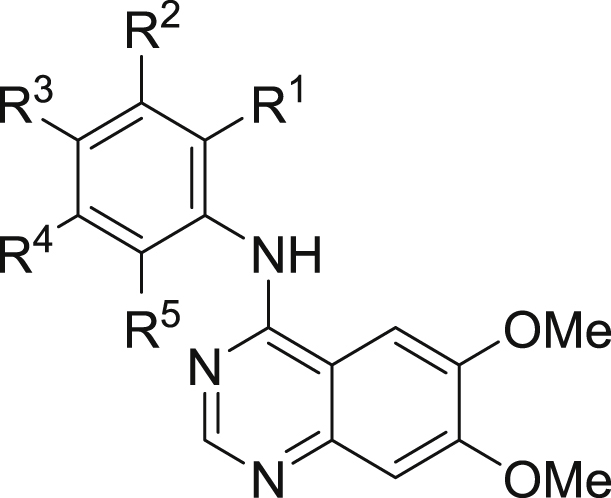
Cellular data for selected anilinoquinazolines.^a^

Cpd	Ar					RET IC_50_ (nM)	KDR IC_50_ (nM)	Cell selectivity ratio	Biochemical selectivity ratio
R^1^	R^2^	R^3^	R^4^	R^5^
**1**	n/a					400 (160)	630 (190)	1	3
**2**	n/a					190 (160)	14 (3)	<1	<1
**4**	F	H	Br	H	H	1700 (290)	2900 (410)	2	5
**6**	H	OH	H	H	H	380 (150)	890 (27)	2	15
**10**	OH	OH	H	H	H	1100 (160)	5700 (710)	5	590
**11**	Br	OH	H	H	H	2400 (380)	>10000	≥4	178
**12**	Cl	OH	H	H	H	1900 (430)	>10000	≥5	132
**13**	Me	OH	H	H	H	6300 (870)	>10000	≥2	≥50
**14**	F	OH	H	H	H	570 (87)	1300 (220)	2	28
**19**	H	OH	F	H	H	490 (140)	360 (88)	<1	23
**20**	H	OH	Me	H	H	500 (77)	88 (7.4)	<1	4
**21**	H	OH	Cl	H	H	940 (420)	110 (15)	<1	6
**22**	H	OH	OMe	H	H	1900 (57)	310 (64)	<1	7
**23**	H	OH	H	F	H	200 (65)	260 (49)	1	25
**24**	H	OH	H	Me	H	1500 (280)	1200 (240)	<1	12
**25**	H	OH	H	H	F	53 (9)	98 (14)	2	18
**26**	H	OH	H	H	Cl	85 (29)	240 (64)	3	27
**27**	H	OH	H	H	Me	460 (100)	1400 (100)	3	19
**28**	H	OH	F	H	F	53 (12)	66 (8.1)	1	23
**29**	H	OH	Cl	H	Cl	1400 (430)	280 (79)	<1	3
**30**	H	OH	Me	H	F	41 (13)	9.5 (5.8)	<1	3
**32**	F	OH	F	H	H	2000 (170)	4700 (1100)	2	115
**33**	F	OH	H	H	F	120 (21)	770 (95)	6	41
**34**	F	OH	H	H	Cl	270 (24)	1900 (160)	7	48
**35**	Me	OH	H	F	H	2100 (1210)	>10000	≥5	281
**36**	Me	OH	H	H	F	2100 (260)	>10000	≥5	130
**37**	Me	OH	H	H	Cl	3600 (1100)	>10000	≥3	≥83
**38**	Cl	OH	Me	H	H	460 (170)	1000 (210)	2	39
**39**	Cl	OH	H	H	Cl	2200 (190)	>10000	≥5	144
**40**	Cl	OH	H	H	F	260 (25)	5400 (1100)	21	282

a Most of these data are expressed as the geometric mean of at least four independent determinations and standard deviations are quoted in parentheses.

**Table 3:**
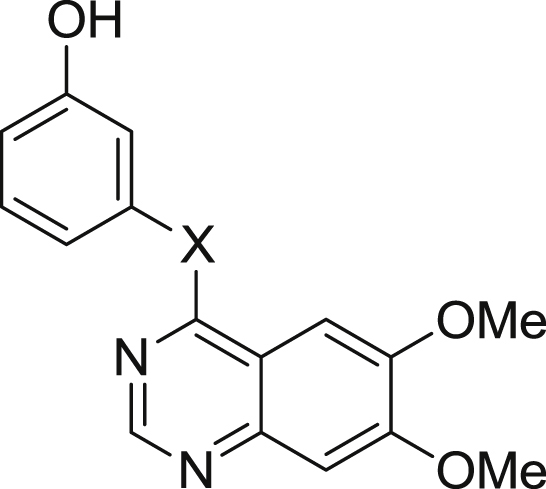
Biochemical and cellular data for alternative linkers.

Cpd	X	Biochemical data^a^			Cellular data^b^		
RET IC_50_ (nM)	KDR IC_50_ (nM)	Selectivity	RET IC_50_ (nM)	KDR IC_50_ (nM)	Selectivity
**41**	NMe	210 (58)	4200 (990)	20	7200 (560)	>10000	≥1
**42**	O	16 (0.28)	150 (62)	9	620 (95)	840 (130)	1
**43**	S	23 (0.42)	210 (10)	9	2000 (190)	1500 (83)	<1

a Biological values are expressed as the geometric mean of four independent determinations and standard deviations are quoted in parentheses.

b Most of these data are expressed as the geometric mean of at least four independent determinations and standard deviations are quoted in parentheses.

**Table 4:**
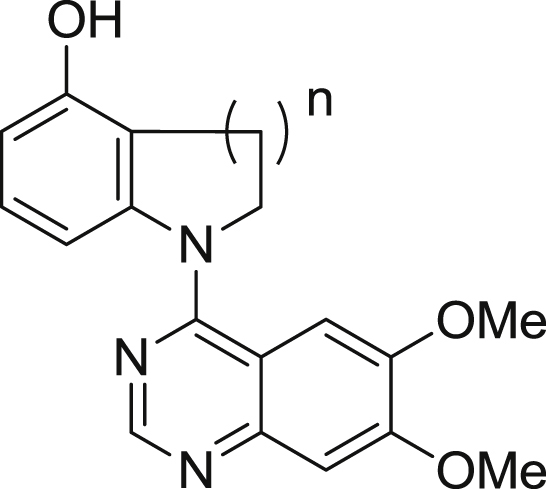
Biochemical and cellular data for tethered compounds.

Cpd	n	Biochemical data^a^			Cellular data^b^		
RET IC_50_ (nM)	KDR IC_50_ (nM)	Selectivity	RET IC_50_ (nM)	KDR IC_50_ (nM)	Selectivity
**44**	1	60 (12)	9500 (1100)	158	4940 (1020)	>10000	≥2
**45**	2	4100 (110)	>10000	≥2	nd	nd	nd

a Biological values are expressed as the geometric mean of four independent determinations and standard deviations are quoted in parentheses.

b Most of these data are expressed as the geometric mean of at least four independent determinations and standard deviations are quoted in parentheses.

**Table 5:**
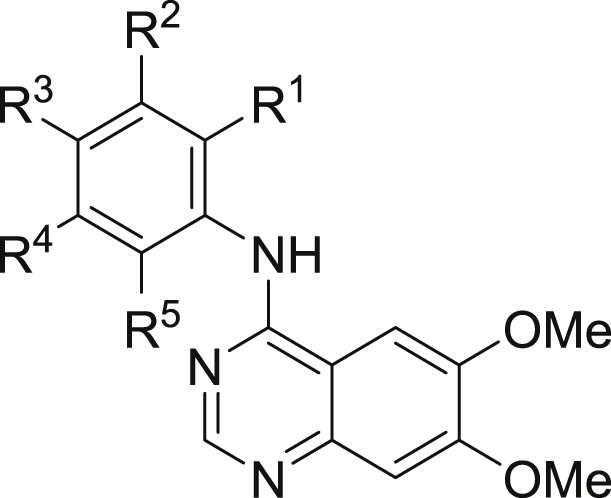
Further profiling of selected quinazolines.

Cpd	Ar					CL_int_ (μL/min/10^6^ cells)	Predicted p*K*_a_^a^	XLogP^b^	Cytotoxicity IC_50_ (nM)^c^
R^1^	R^2^	R^3^	R^4^	R^5^
**6**	H	OH	H	H	H	76.9	9.6	2.78	>10000
**10**	OH	OH	H	H	H	95.5	9.3	2.41	>10000
**11**	Br	OH	H	H	H	157	8.0	3.58	>10000
**12**	Cl	OH	H	H	H	174	8.1	3.40	>10000
**13**	Me	OH	H	H	H	28.4	9.9	3.06	>10000
**30**	H	OH	Me	H	F	91.9	10.0	3.16	>1000
**33**	F	OH	H	H	F	68.9	8.3	2.98	>10000
**34**	F	OH	H	H	Cl	93.3	7.9	3.50	>10000
**35**	Me	OH	H	F	H	54.8	8.9	3.16	>10000
**36**	Me	OH	H	H	F	20.9	10.0	3.16	>10000
**40**	Cl	OH	H	H	F	66.7	8.1	3.50	>10000

a Calculated using ACD/Percepta, 2012 release (build 2254), Advanced Chemistry Development, Inc., Toronto, ON, Canada, www.acdlabs.com.

b Calculated using Dotmatics software suite, Dotmatics, Bishops Stortford, Herts, UK, www.dotmatics.com.

c Cell cytotoxicity was measured in a matched wild-type (non RET-driven) BaF3 cell line.

## Appendix A Supplementary data

Supporting information available: LC-MS methods and solvent gradients, preparative HPLC instrument and solvent gradients, summary of purity data for final compounds, NMR and high-resolution mass spectra for representative key derivatives, structure determination protocols and detailed structural description of **6** bound to the RET kinase domain. PDB Accession Code 5AMN.
