# Supplementary material for: The discovery of 2-substituted phenol quinazolines as potent RET kinase inhibitors with improved KDR selectivity
Source: Eur J Med Chem. 2016 Apr 13;112:20–32. doi: 10.1016/j.ejmech.2016.01.039 (PMC4896931; doi:10.1016/j.ejmech.2016.01.039)
Supplement: Supplementary file 1 [file mmc1.docx]

**SUPPLEMENTARY DATA**

The Discovery of 2-Substituted Phenol Quinazolines as Potent RET Kinase Inhibitors with Improved KDR Selectivity

Rebecca Newton,^*,†^ Katherine A. Bowler,^†^ Emily M. Burns,^§^ Philip J. Chapman,^†^ Emma E. Fairweather,^†^ Samantha J. R. Fritzl,^†^ Kristin M. Goldberg,^†^ Niall M. Hamilton,^†^ Sarah V. Holt,^†^ Gemma V. Hopkins,^†^ Stuart D. Jones,^†^ Allan M. Jordan,^†^ Amanda J. Lyons,^†^ H. Nikki March,^†^ Neil Q. McDonald,^§,‡^ Laura A. Maguire,^†^ Daniel P. Mould,^†^ Andrew G. Purkiss,^§^ Helen F. Small,^†^ Alexandra I. J. Stowell,^†^ Graeme J. Thomson,^†^ Ian D. Waddell,^†^ Bohdan Waszkowycz,^†^ Amanda J. Watson^†^ and Donald J. Ogilvie^†^

^†^Cancer Research UK Manchester Institute, Drug Discovery Unit, University of Manchester, Wilmslow Road, Withington, Manchester, M20 4BX, England

^§^Structural Biology Laboratory, Cancer Research UK London Research Institute, London, WC2A 3LY, England

^‡^Institute of Structural and Molecular Biology, Department of Biological Sciences,

Birkbeck College, Malet Street, London WC1E 7HX, England

**Table of Contents**

*LC-MS methods and solvent gradients:* S2

*Preparative HPLC instrument and solvent gradients:* S3

*Summary of purity data for final compounds:* S4

*1H NMR Spectra for representative key derivatives:* S6

*HRMS spectra for representative key derivatives:* S17

*Structure determination protocols:* S27

*Detailed structural description of* ***6*** *bound to RET KD:* S29

*References:* S30

*LC–MS methods and solvent gradients*

LC–MS analyses were performed on a Waters Acquity UPLC system fitted with BEH C18 1.7 µM columns (2.1 × 50 mm) and with a UV detector. Positive and negative mass ion detection was performed using a Waters SQD detector. Analyses were performed with either buffered acidic or basic solvents and gradients as detailed below:

Low pH:

Solvent A – Water + 10 mM ammonium formate + 0.1% formic acid

Solvent B – Acetonitrile + 5% water + 0.1% formic acid

High pH:

Solvent A – Water + 10 mM ammonium hydrogen carbonate + 0.1% ammonia solution

Solvent B – Acetonitrile + 0.1% ammonia solution

Gradient:

| Time | Flow rate  (mL min^-1^) | % Solvent A | % Solvent B |
| --- | --- | --- | --- |
| 0 | 0.6 | 95 | 5 |
| 1.2 | 0.6 | 5 | 95 |
| 1.7 | 0.6 | 5 | 95 |
| 1.8 | 0.6 | 95 | 5 |

*Preparative HPLC instrument and solvent gradients*

Several compounds were purified by preparative HPLC on a Waters FractionLynx MS autopurification system, with a Waters XBridge 5 DM C18, 100 mm × 19 mm i.d. column, running at a flow rate of 20 mL min^-1^ with UV diode array detection (210–400 nm) and mass-directed collection using both positive and negative mass ion detection. Purifications were performed using acidic or basic solvent systems as appropriate. Compound retention times on the system were routinely assessed using a 30–50 µL test injection and a standard gradient, and then purified using an appropriately selected focussed gradient as detailed below, based upon observed retention time.

Low pH:

Solvent A – Water + 10 mM ammonium formate + 0.1% formic acid

Solvent B – Acetonitrile + 5% water +0.1% formic acid

High pH:

Solvent A – Water + 10 mM ammonium formate + 0.1% ammonia solution

Solvent B – Acetonitrile + 5% water + 0.1% ammonia solution

Standard Gradient:

| Time | Flow rate  (mL min^-1^) | % Solvent A | % Solvent B |
| --- | --- | --- | --- |
| 0 | 20 | 90 | 10 |
| 0.3 | 20 | 90 | 10 |
| 8.5 | 20 | 2 | 98 |
| 12 | 20 | 2 | 98 |
| 12.5 | 0 | 2 | 98 |

Focused Gradients:

| Time | Flow rate  (mL min-1) | % Solvent B | | | | |
| --- | --- | --- | --- | --- | --- | --- |
|  |  | Retention time on standard gradient (min.) | | | | |
|  |  | 0–5.2 | 4.9–6.6 | 6.3–7.5 | 7.3–9.5 | 9.3–12 |
| 0 | 20 | 10 | 10 | 10 | 10 | 10 |
| 0.25 | 20 | 10 | 10 | 10 | 10 | 10 |
| 0.35 | 20 | 10 | 20 | 35 | 45 | 60 |
| 10 | 20 | 45 | 55 | 65 | 75 | 98 |
| 12 | 20 | 98 | 98 | 98 | 98 | 98 |
| 12.5 | 0 | 98 | 98 | 98 | 98 | 98 |

*Summary of purity data for final compounds* ***4****-****45***

| **Compound** | **LC-MS pH 4** | | | | **LC-MS pH 10** | | | |
| --- | --- | --- | --- | --- | --- | --- | --- | --- |
|  | **RT** | **Obs MW** | **Adduct** | **Purity** | **RT** | **Obs MW** | **Adduct** | **Purity** |
| **4** | 0.91 | 378 | [M+H]+ | >95 | 1.09 | 378 | [M+H]+ | >95 |
| **5** | 0.69 | 298.6 | [M+H]+ | >95 | 0.83 | 298.6 | [M+H]+ | 90-95 |
| **6** | 0.67 | 298.6 | [M+H]+ | >95 | 0.79 | 298.5 | [M+H]+ | >95 |
| **7** | 0.65 | 298.6 | [M+H]+ | >95 | 0.75 | 298.6 | [M+H]+ | 90-95 |
| **8** | 0.61 | 297.5 | [M+H]+ | >95 | 0.79 | 297.5 | [M+H]+ | >95 |
| **9** | 0.95 | 312.5 | [M+H]+ | >95 | 0.8 | 312.5 | [M+H]+ | >95 |
| **10** | 0.65 | 314.5 | [M+H]+ | >95 | 0.85 | 314.5 | [M+H]+ | >95 |
| **11** | 0.68 | 378.2 | [M+H]+ | >95 | 0.77 | 376.3 | [M+H]+ | 90-95 |
| **12** | 0.68 | 332.5 | [M+H]+ | >95 | 0.72 | 332.5 | [M+H]+ | >95 |
| **13** | 0.64 | 312.5 | [M+H]+ | >95 | 0.74 | 312.5 | [M+H]+ | >95 |
| **14** | 0.65 | 316.1 | [M+H]+ | >95 | 0.74 | 316.5 | [M+H]+ | >95 |
| **15** | 0.65 | 328 | [M+H]+ | >95 | 0.81 | 328 | [M+H]+ | >95 |
| **16** | nd | nd | nd | nd | 0.86 | 326.4 | [M+H]+ | 90-95 |
| **17** | 0.74 | 366.3 | [M+H]+ | >95 | 0.78 | 366.3 | [M+H]+ | 85-90 |
| **18** | 0.72 | 323.5 | [M+H]+ | >95 | 0.78 | 323.5 | [M+H]+ | >95 |
| **19** | 0.68 | 316.5 | [M+H]+ | >95 | 0.81 | 316.6 | [M+H]+ | >95 |
| **20** | 0.74 | 312.5 | [M+H]+ | >95 | 0.93 | 312.5 | [M+H]+ | >95 |
| **21** | 0.76 | 332.5 | [M+H]+ | >95 | 0.88 | 332.5 | [M+H]+ | >95 |
| **22** | 0.67 | 328.6 | [M+H]+ | >95 | 0.82 | 328.6 | [M+H]+ | >95 |
| **23** | 0.71 | 316.5 | [M+H]+ | >95 | 0.88 | 316.5 | [M+H]+ | >95 |
| **24** | 0.71 | 312.5 | [M+H]+ | >95 | 0.88 | 312.5 | [M+H]+ | >95 |
| **25** | 0.69 | 316.5 | [M+H]+ | >95 | 0.81 | 316.5 | [M+H]+ | >95 |
| **26** | 0.73 | 332.5 | [M+H]+ | >95 | 0.87 | 332.5 | [M+H]+ | >95 |
| **27** | 0.67 | 312.6 | [M+H]+ | >95 | 0.81 | 312.6 | [M+H]+ | >95 |
| **28** | 0.71 | 334.6 | [M+H]+ | >95 | 0.76 | 334.5 | [M+H]+ | >95 |
| **29** | 0.82 | 366.5 | [M+H]+ | 85-90 | 0.81 | 366.5 | [M+H]+ | 85-90 |
| **30** | 0.76 | 330.6 | [M+H]+ | >95 | 0.89 | 330.6 | [M+H]+ | >95 |
| **31** | 0.82 | 346.5 | [M+H]+ | >95 | 0.95 | 346.5 | [M+H]+ | >95 |
| **32** | 0.69 | 334.5 | [M+H]+ | 90-95 | 0.62 | 334.5 | [M+H]+ | 90-95 |
| **33** | 0.7 | 334.5 | [M+H]+ | >95 | 0.72 | 334.5 | [M+H]+ | >95 |
| **34** | 0.72 | 350.1 | [M+H]+ | >95 | 0.76 | 350.1 | [M+H]+ | >95 |
| **35** | 0.72 | 330 | [M+H]+ | >95 | 0.85 | 330 | [M+H]+ | >95 |
| **36** | 0.68 | 330.5 | [M+H]+ | >95 | 0.82 | 330.5 | [M+H]+ | >95 |
| **37** | 0.75 | 346.5 | [M+H]+ | >95 | 0.86 | 346.5 | [M+H]+ | >95 |
| **38** | 0.73 | 346.6 | [M+H]+ | >95 | 0.88 | 346.6 | [M+H]+ | 90-95 |
| **39** | 0.75 | 366 | [M+H]+ | >95 | 0.68 | 366 | [M+H]+ | >95 |
| **40** | 0.71 | 350.1 | [M+H]+ | >95 | 0.7 | 350.1 | [M+H]+ | >95 |
| **41** | 0.72 | 312.5 | [M+H]+ | 90-95 | 0.87 | 312.5 | [M+H]+ | 90-95 |
| **42** | 0.91 | 299.5 | [M+H]+ | >95 | 0.91 | 299.5 | [M+H]+ | 95 |
| **43** | 0.97 | 315.5 | [M+H]+ | 85-90 | 0.99 | 315.5 | [M+H]+ | >95 |
| **44** | 0.75 | 324.3 | [M+H]+ | >95 | 0.88 | 324.3 | [M+H]+ | >95 |
| **45** | 0.8 | 338.4 | [M+H]+ | 90-95 | 0.91 | 338.3 | [M+H]+ | >95 |

**1H NMR spectrum for Compound 6:**

**
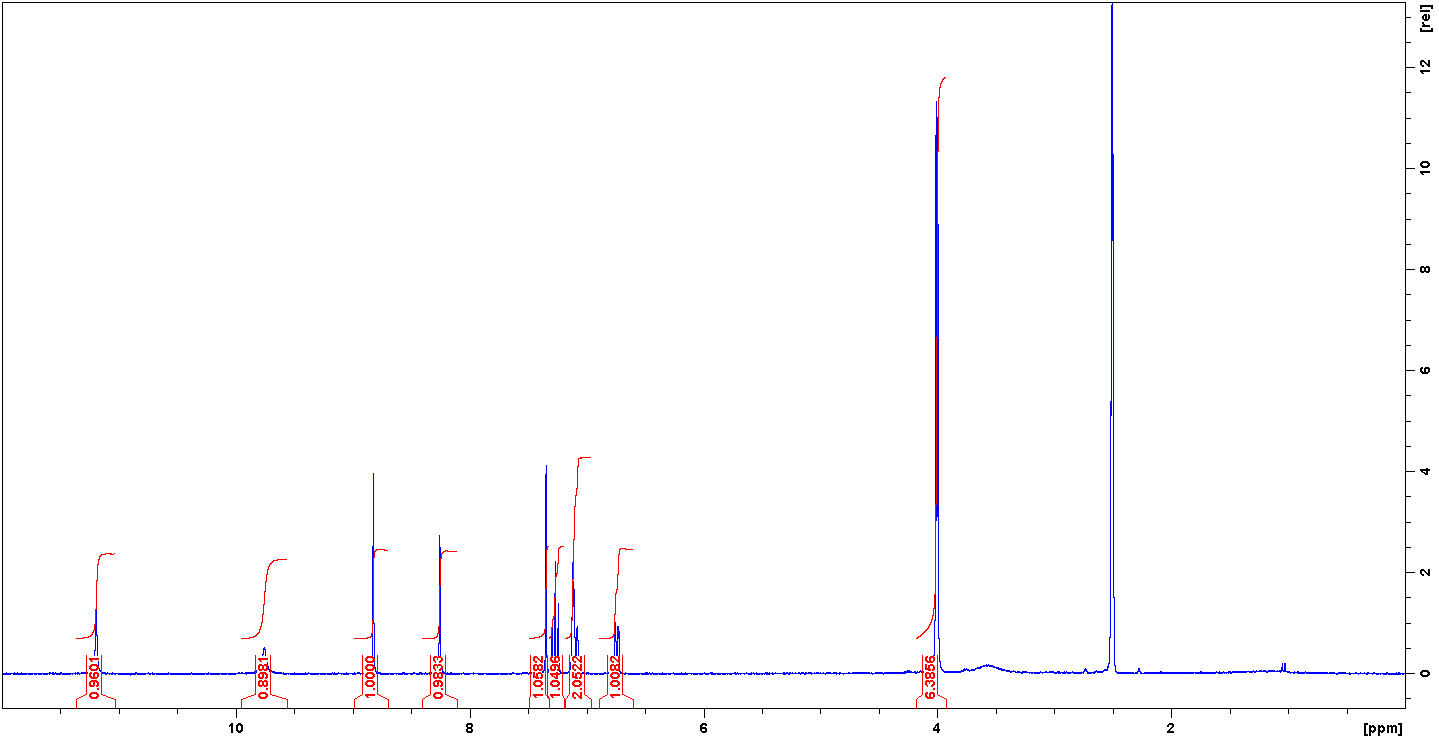
**

**1H NMR spectrum for Compound 10:**

**
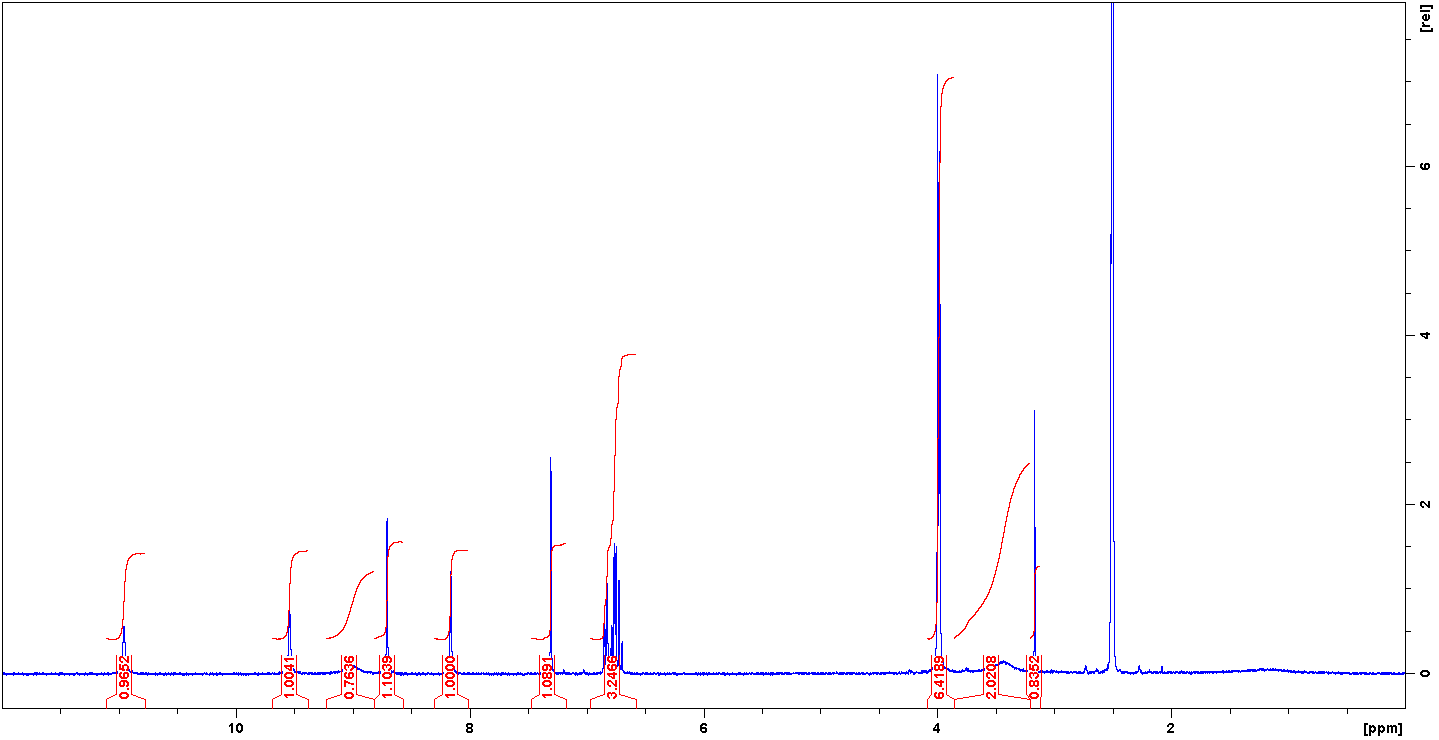
1H NMR spectrum for Compound 11:**

**
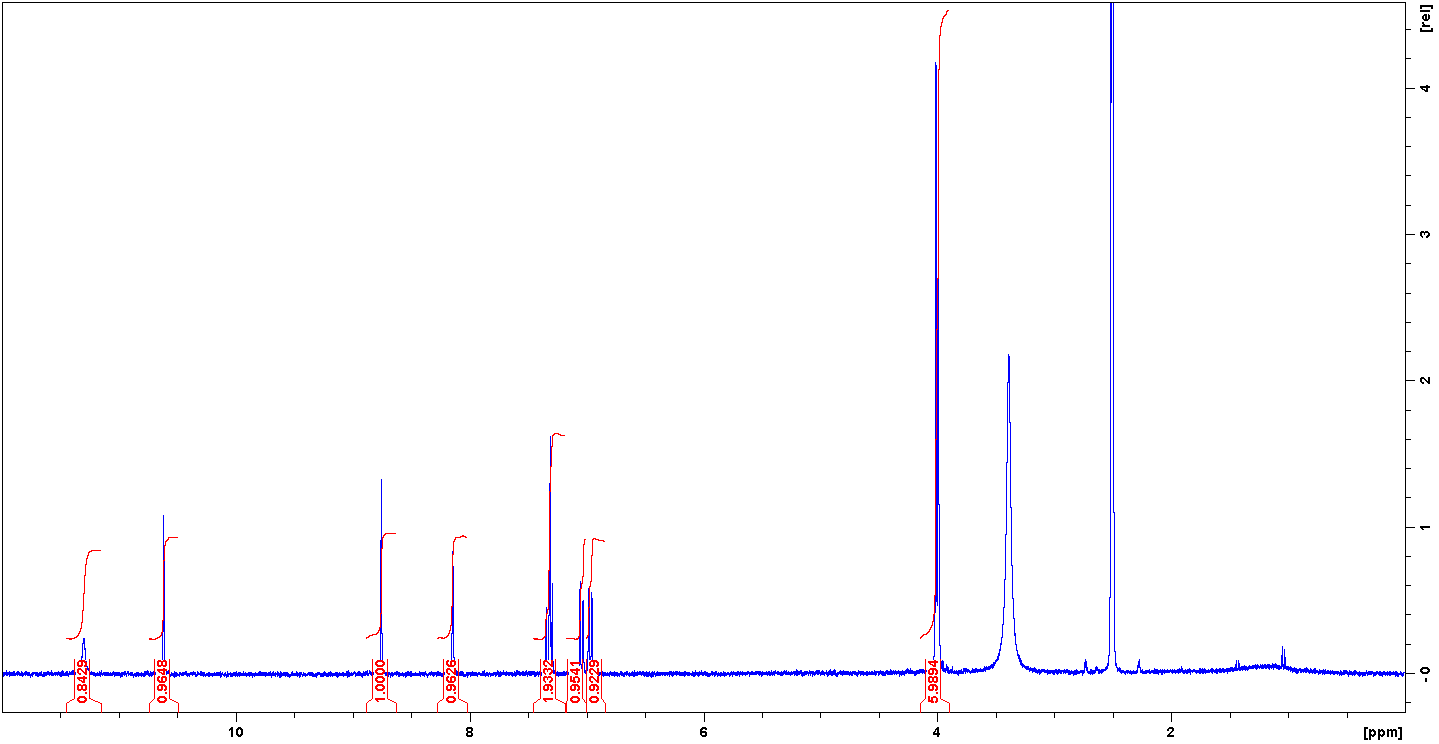
**

**1H NMR spectrum for Compound 12:**

**
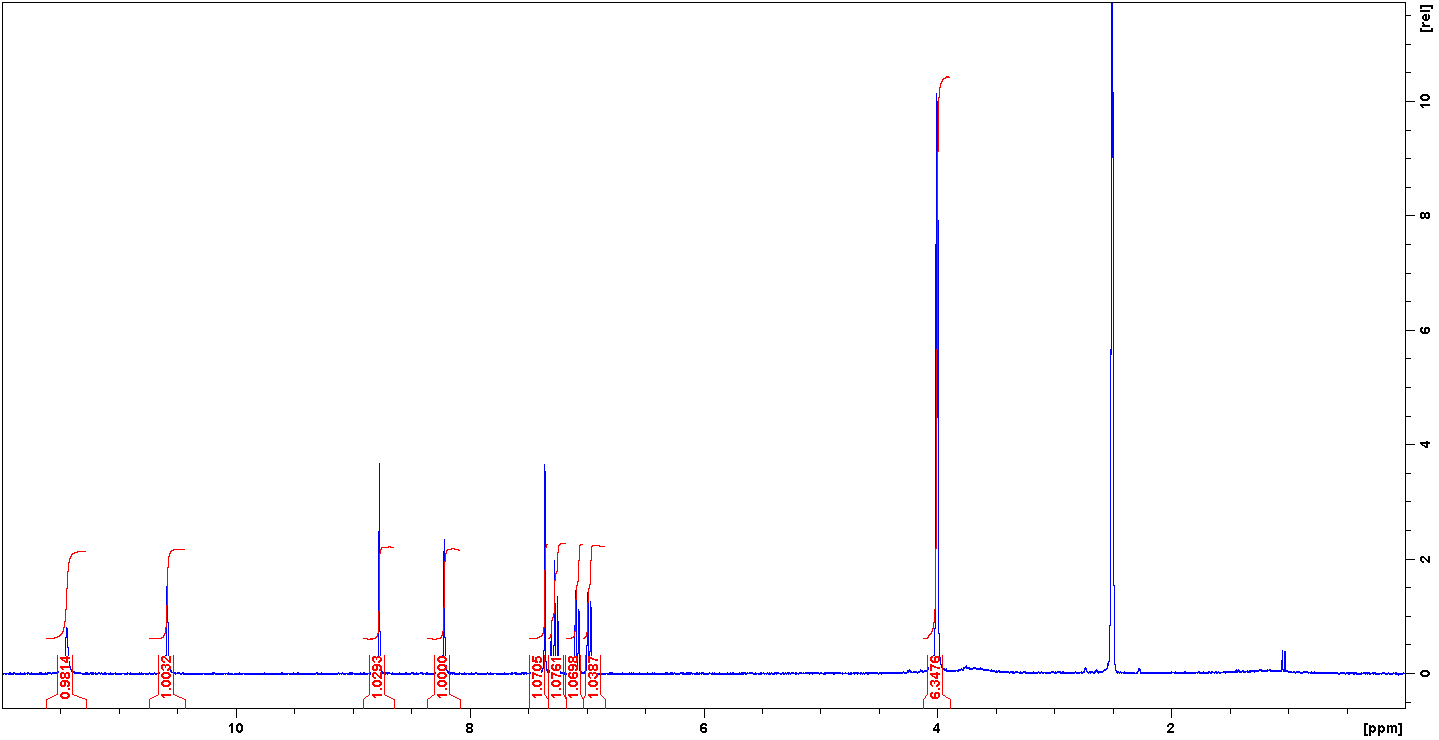
**

**1H NMR spectrum for Compound 13:**

**
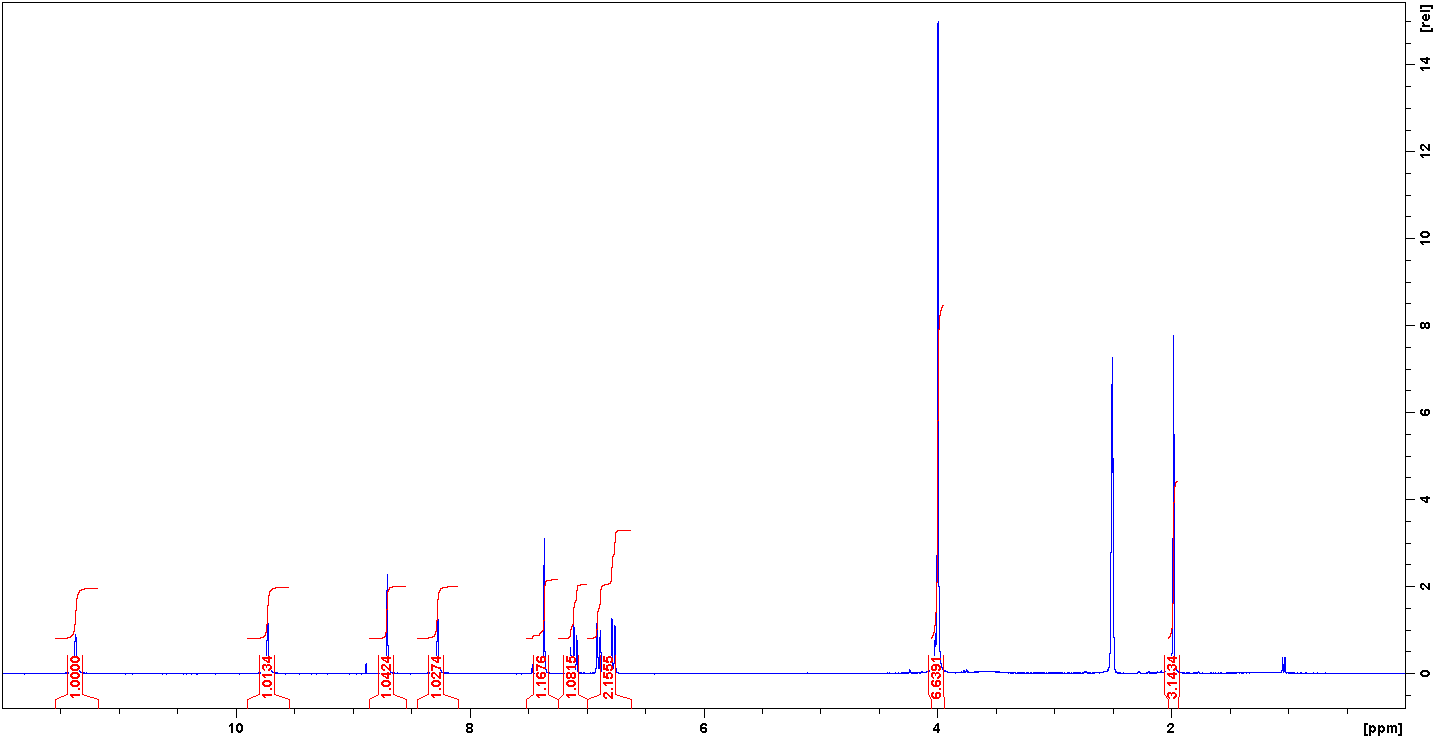
**

**1H NMR spectrum for Compound 30:**

**
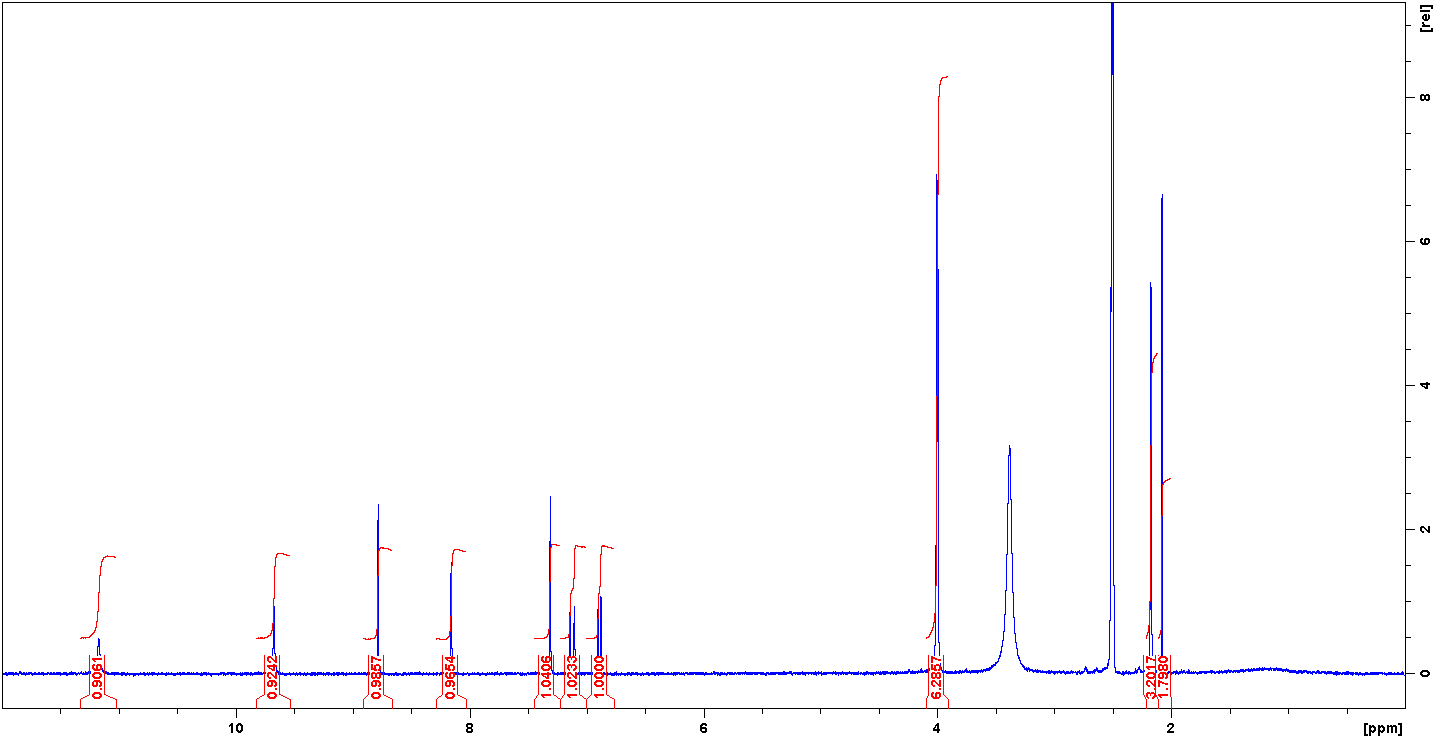
**

**1H NMR spectrum for Compound 33:**

**
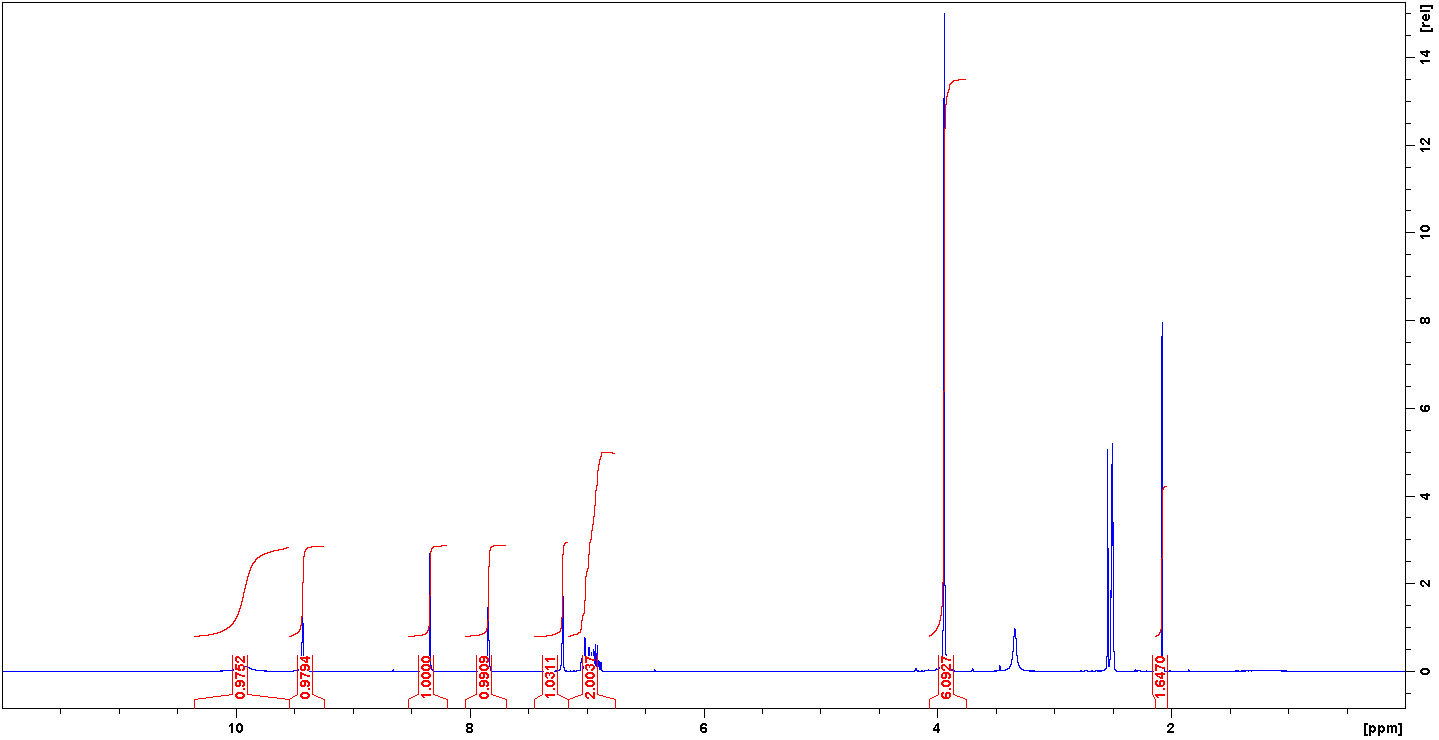
**

**1H NMR spectrum for Compound 34:**

**
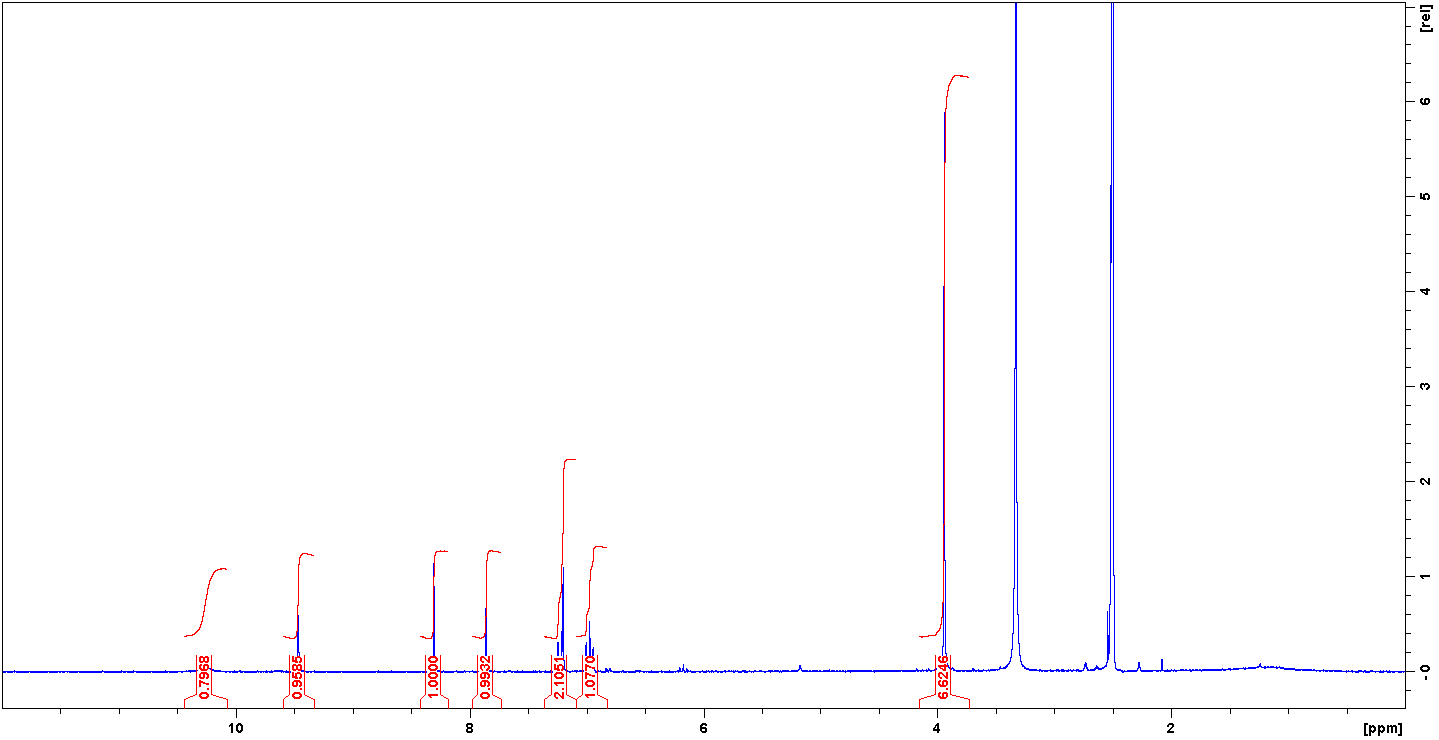
**

**1H NMR spectrum for Compound 35:**

**
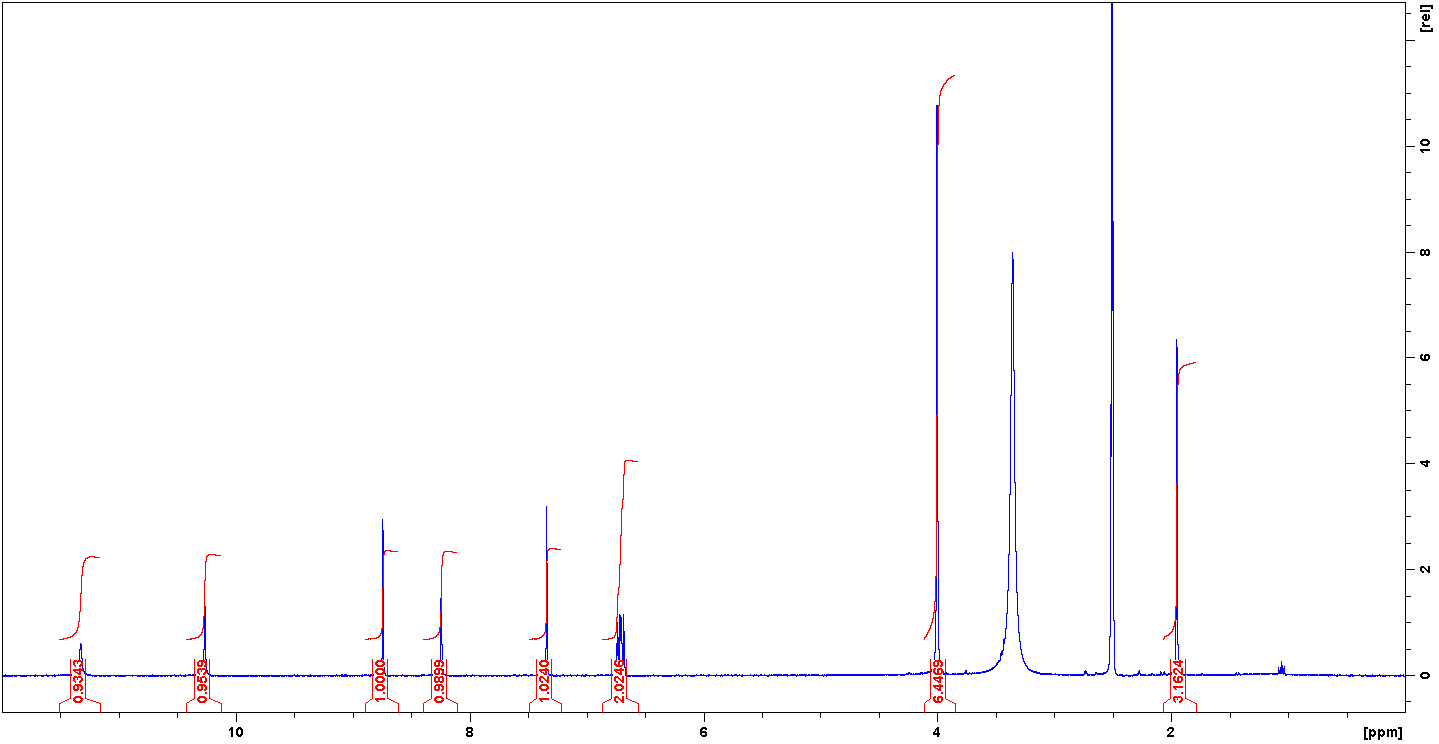
**

**1H NMR spectrum for Compound 36:**

**
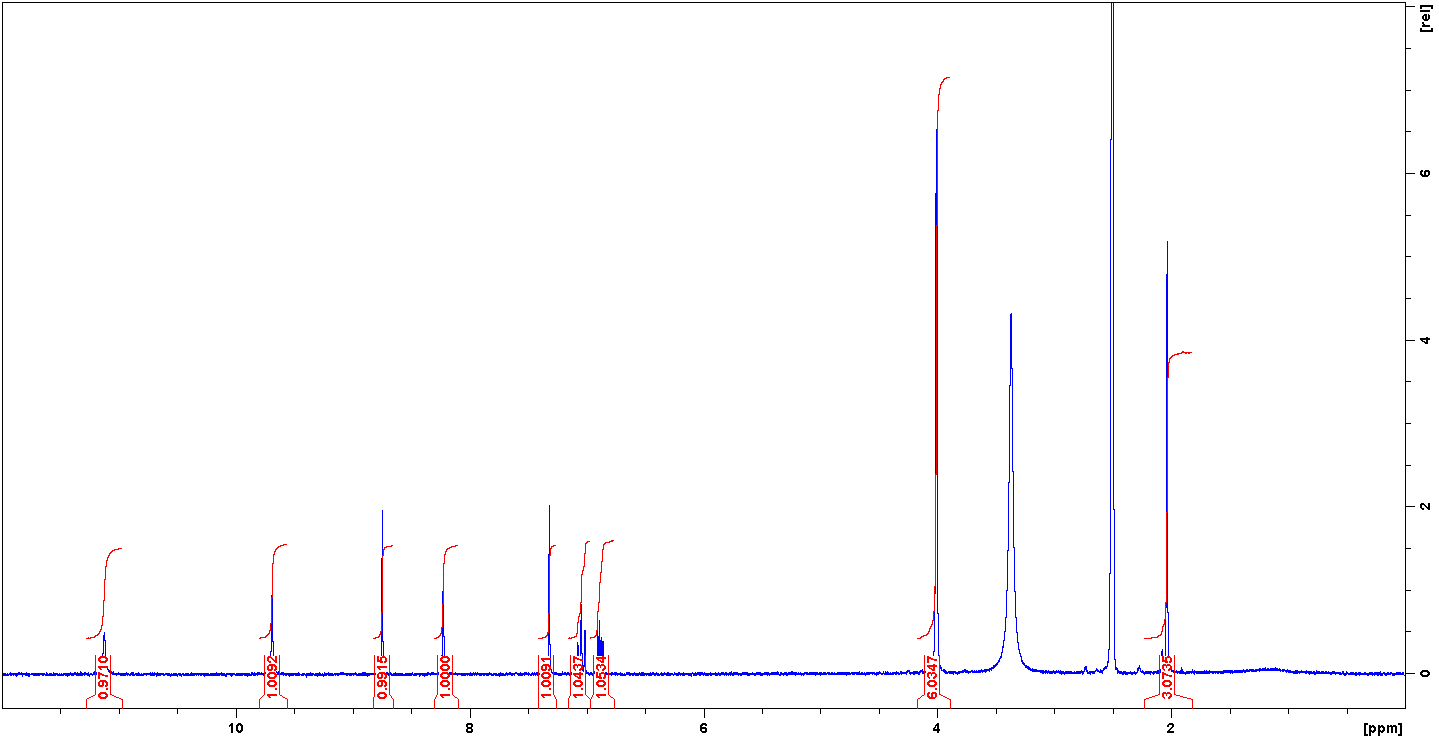
**

**1H NMR spectrum for Compound 40:**

**
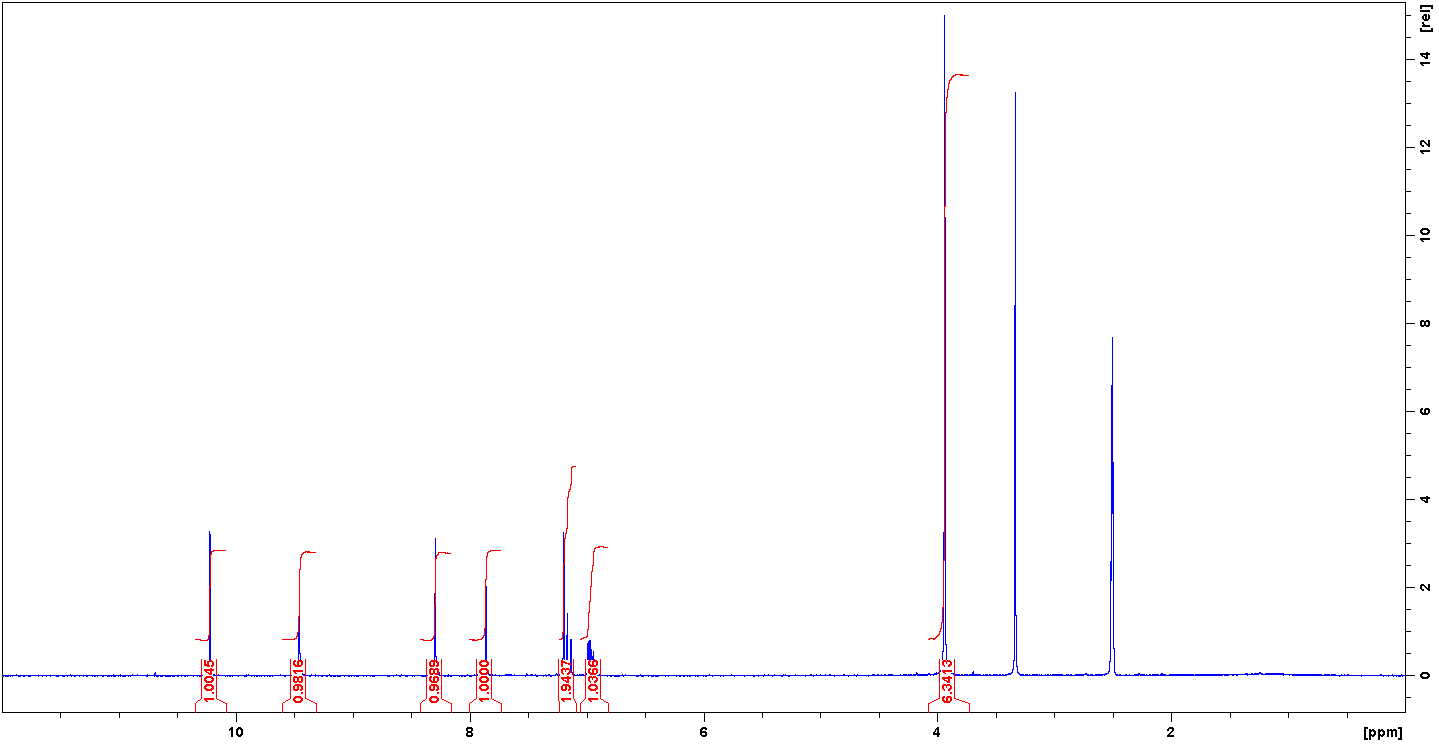
**

**HRMS spectrum for Compound 6:**


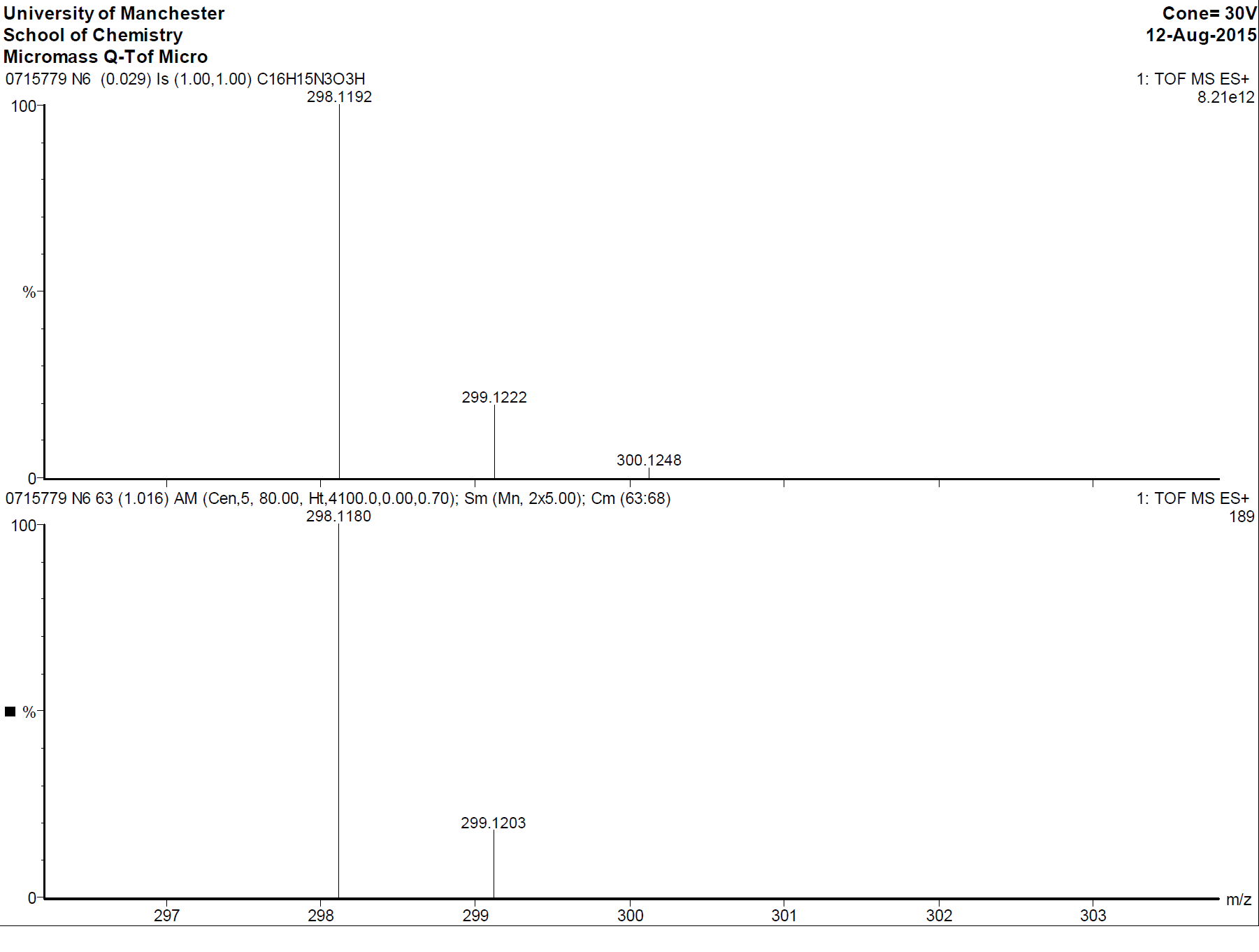


**HRMS spectrum for Compound 10:**


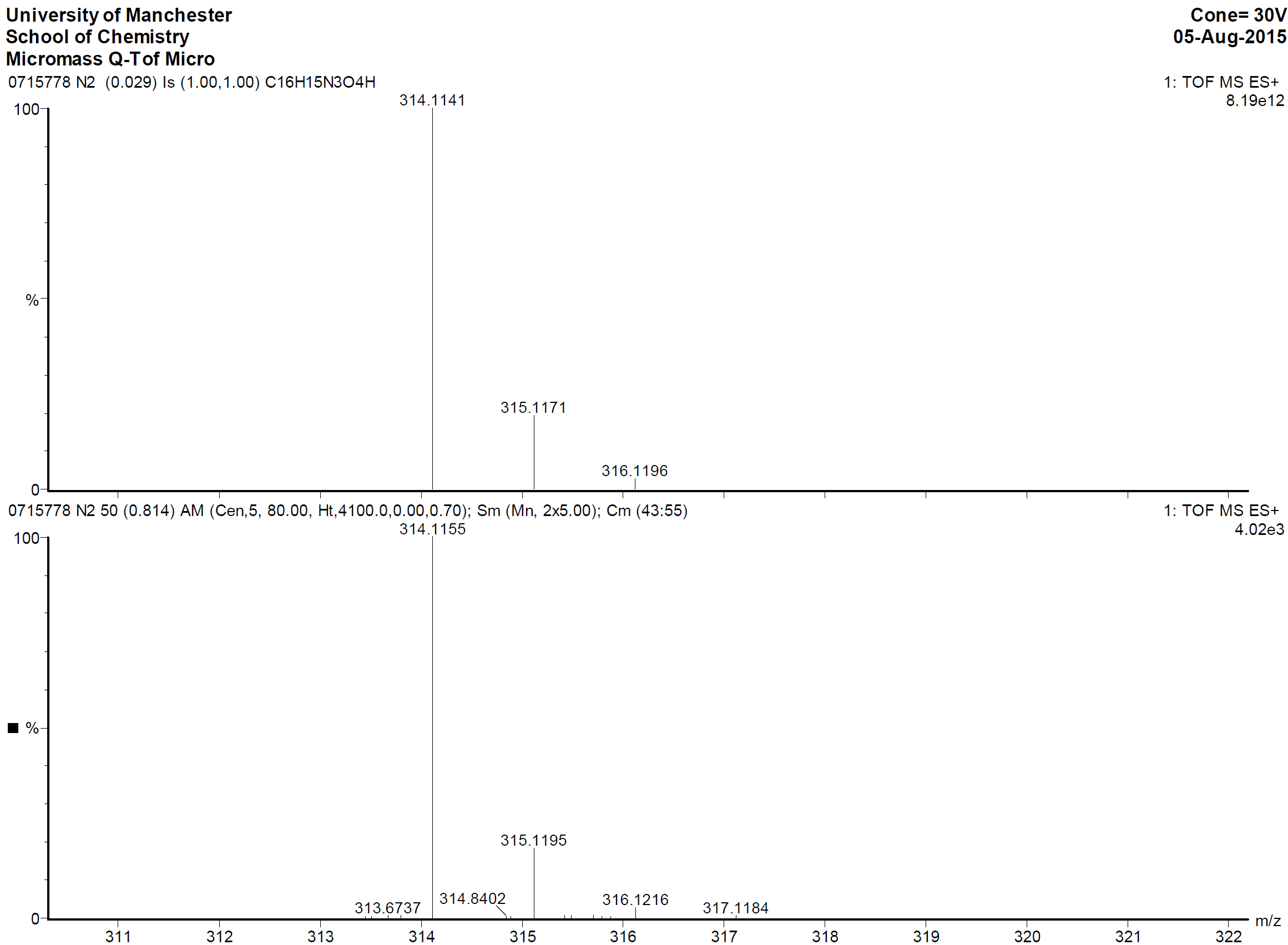


**HRMS spectrum for Compound 11:**


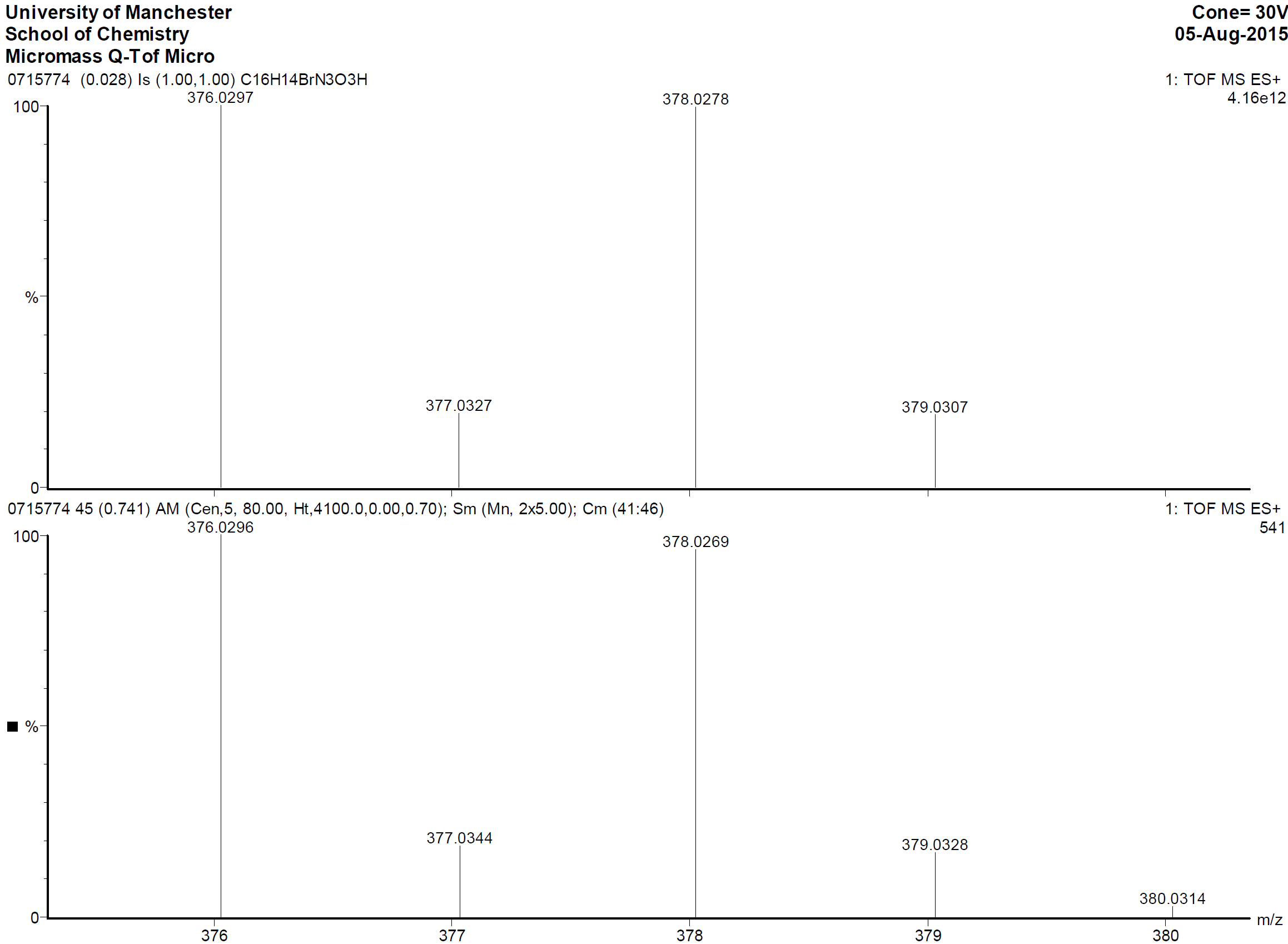


**HRMS spectrum for Compound 12:**


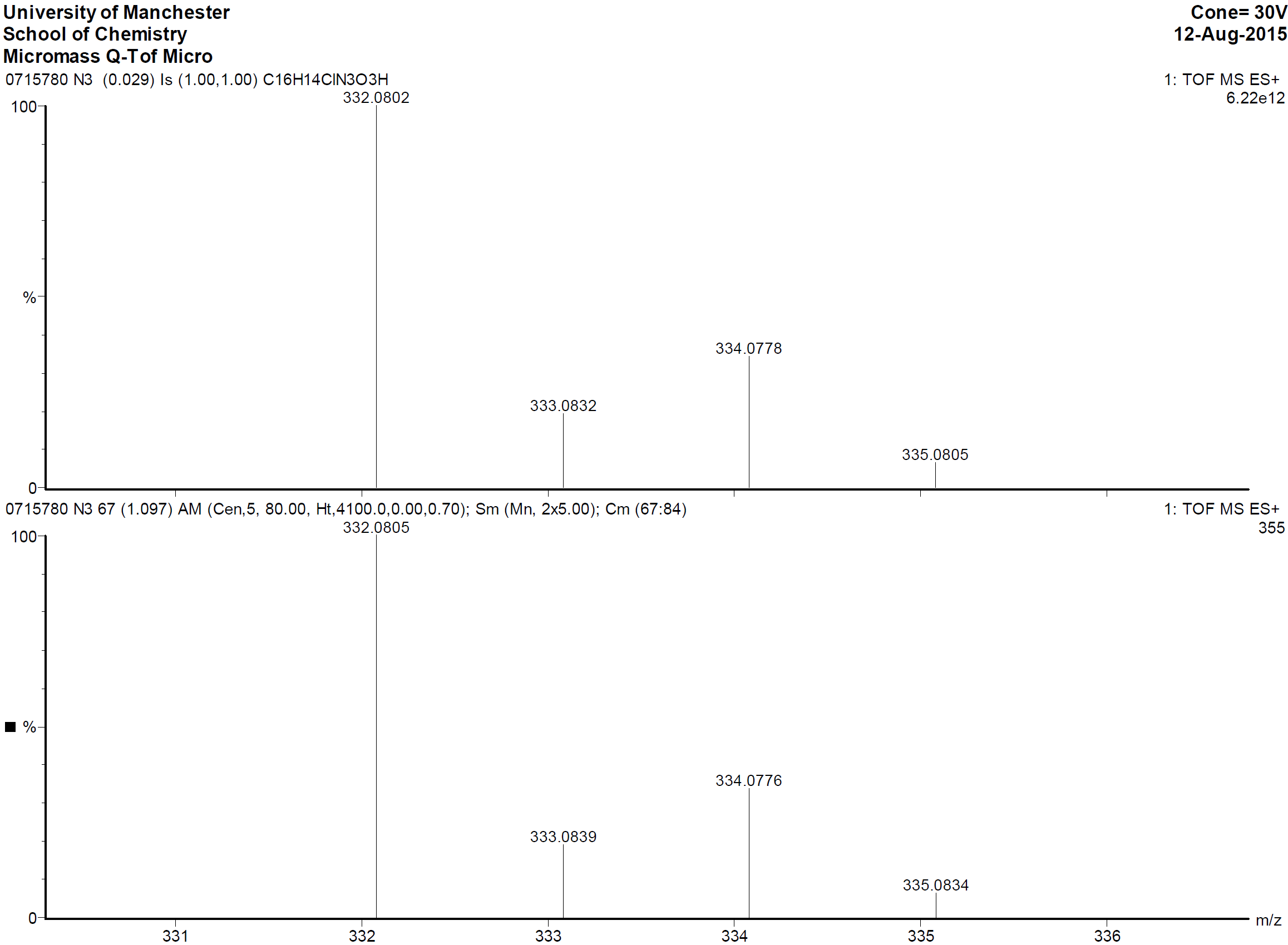


**HRMS spectrum for Compound 13:**


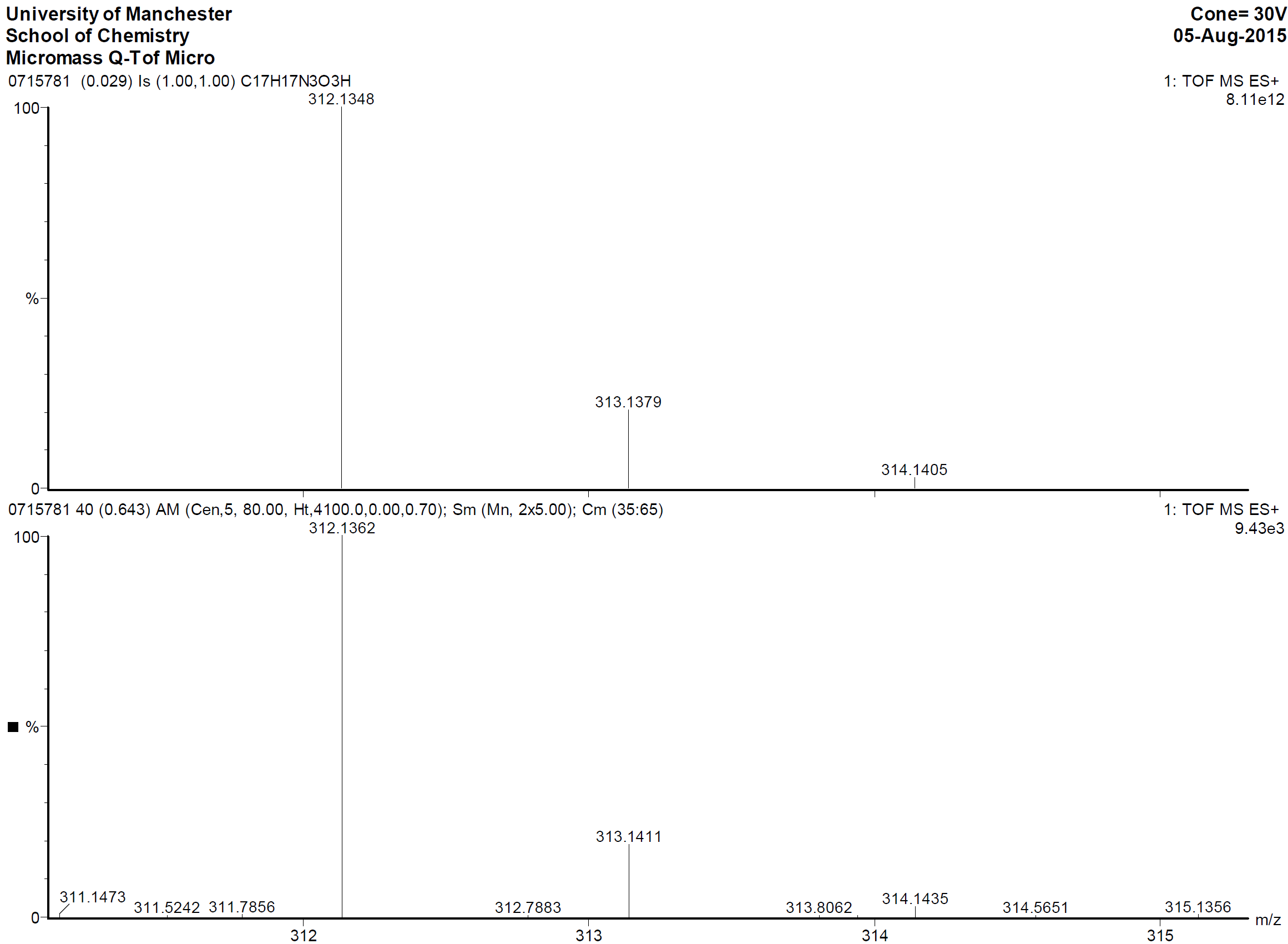


**HRMS spectrum for Compound 30:**


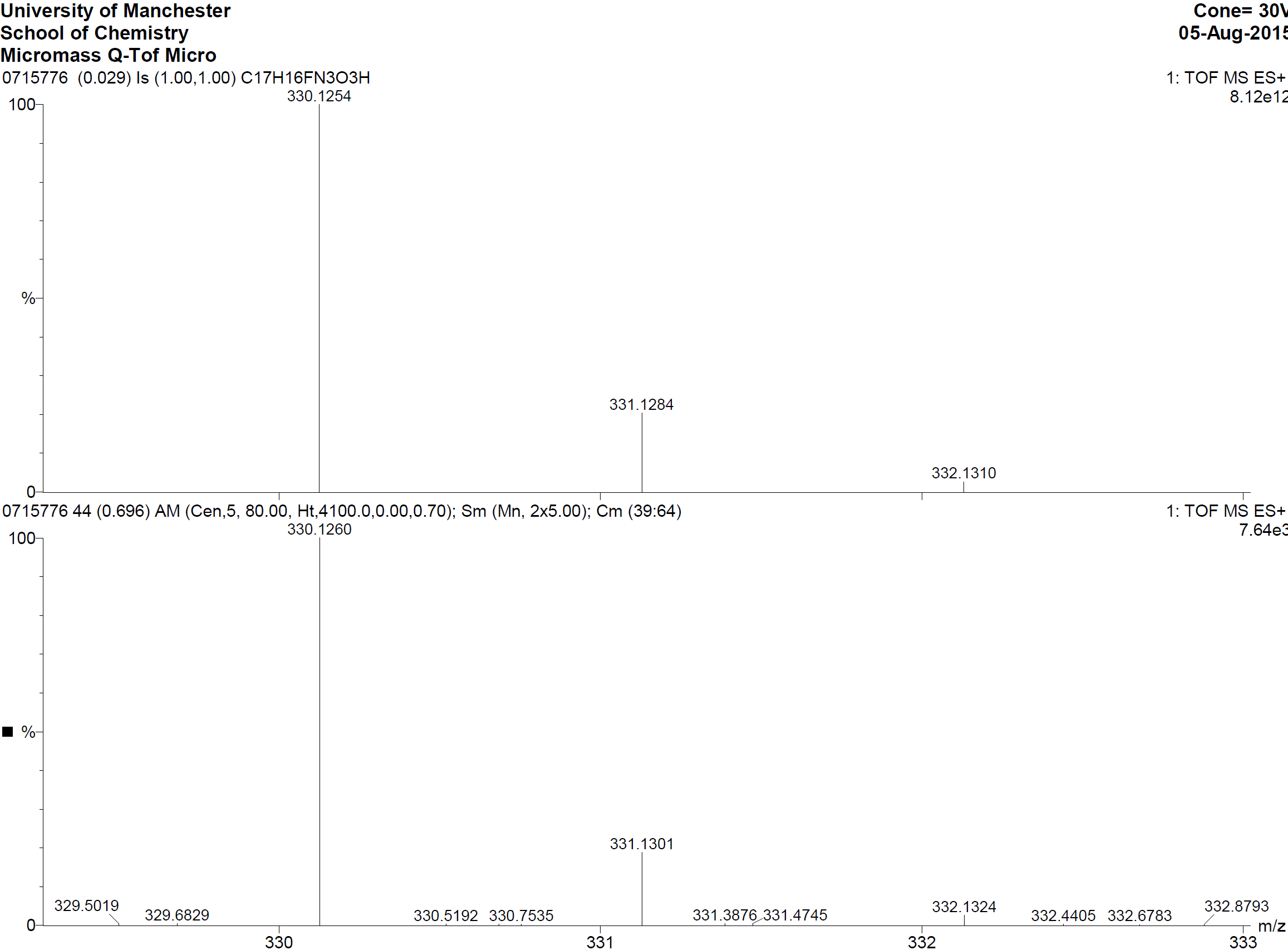


**HRMS spectrum for Compound 33:**


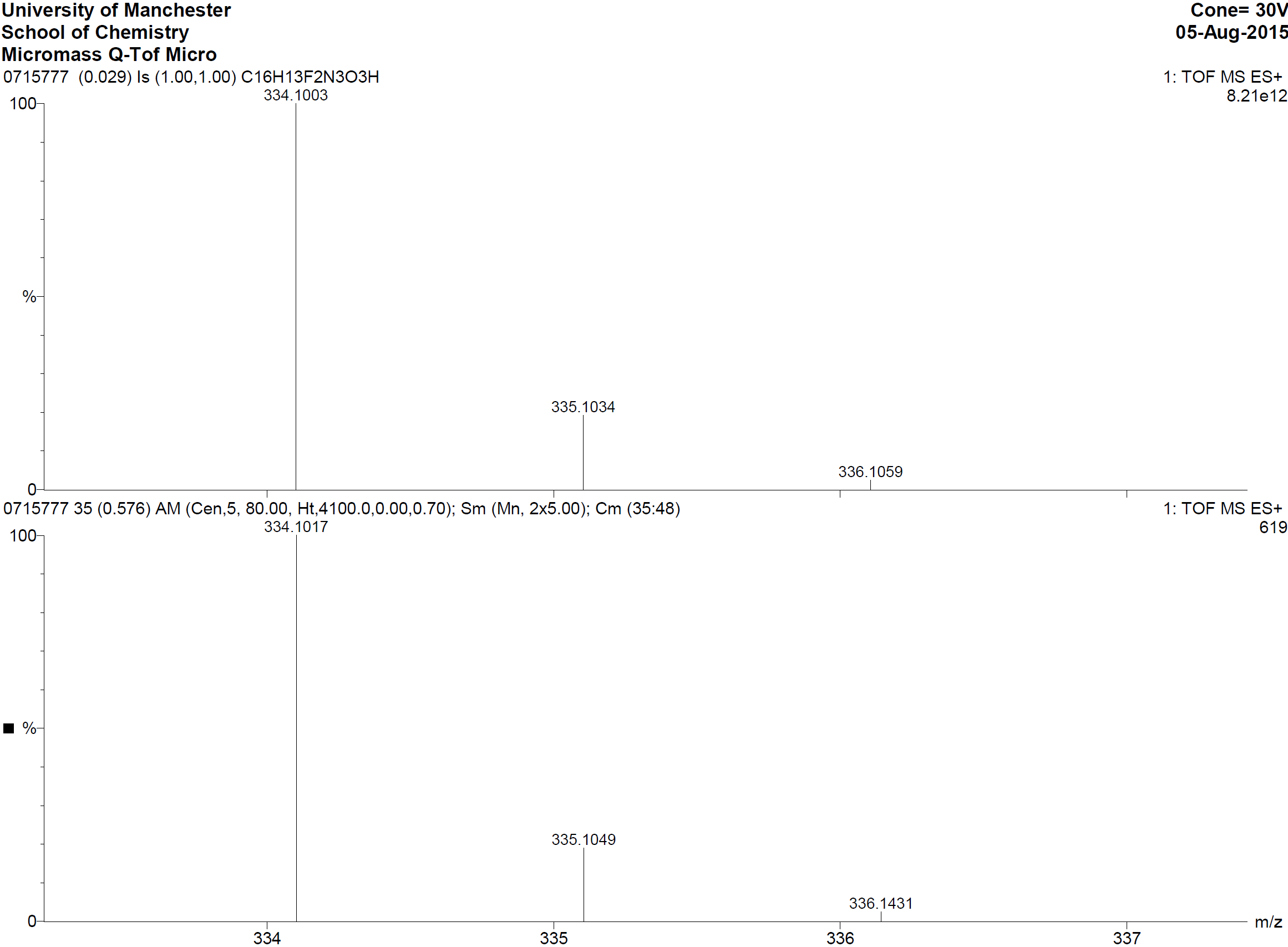


**HRMS spectrum for Compound 34:**


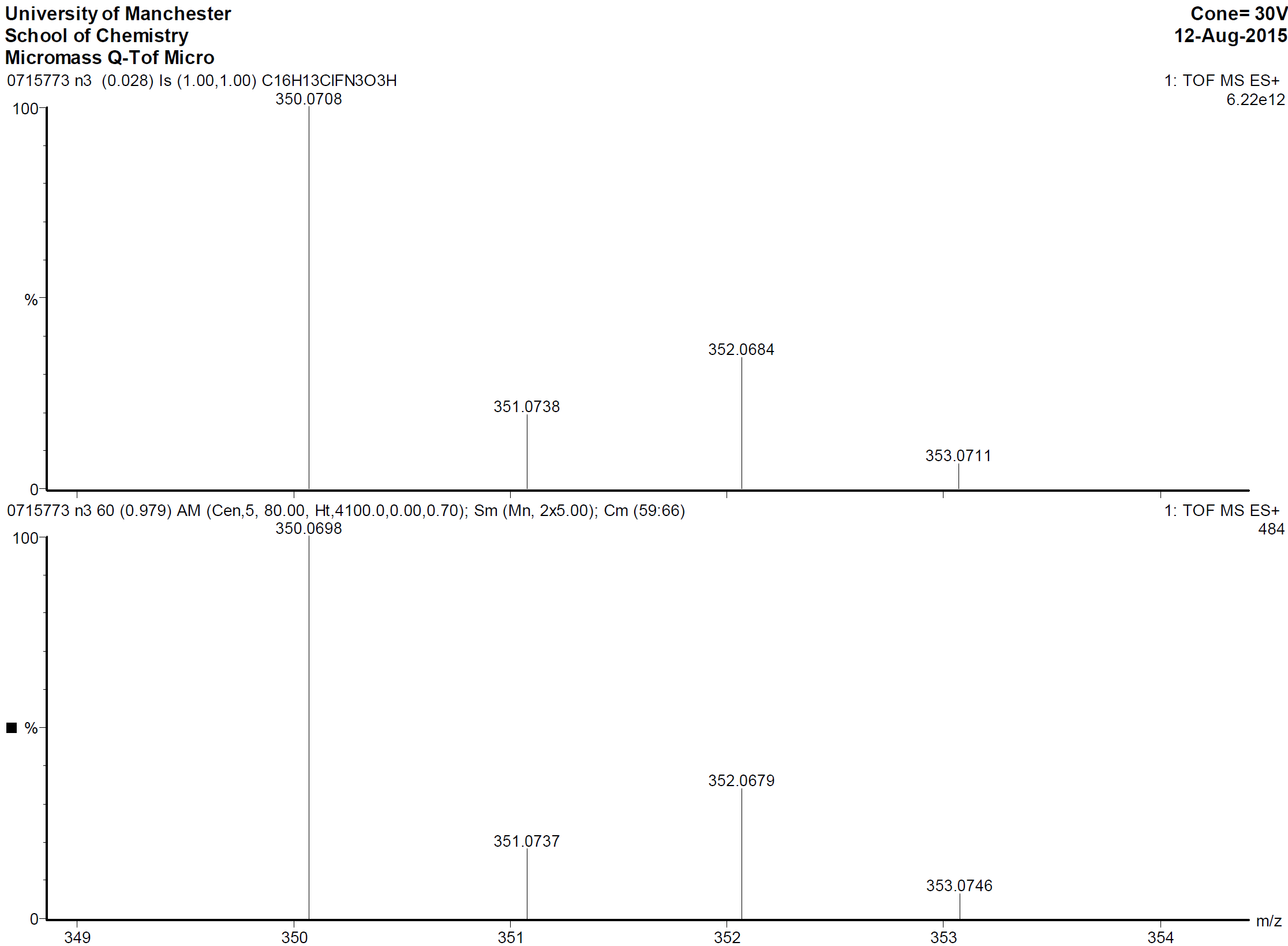


**HRMS spectrum for Compound 36:**


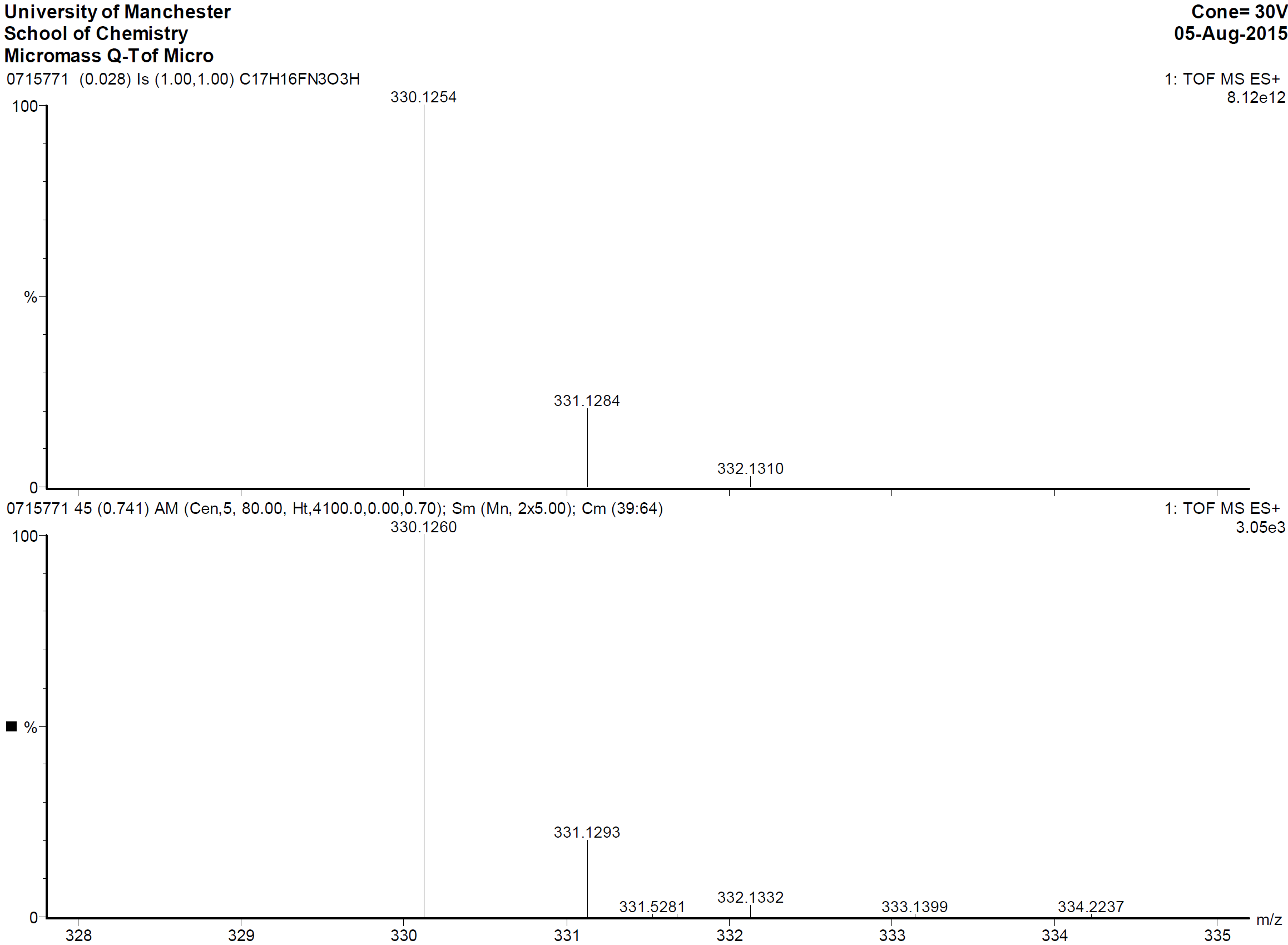


**HRMS spectrum for Compound 40:**


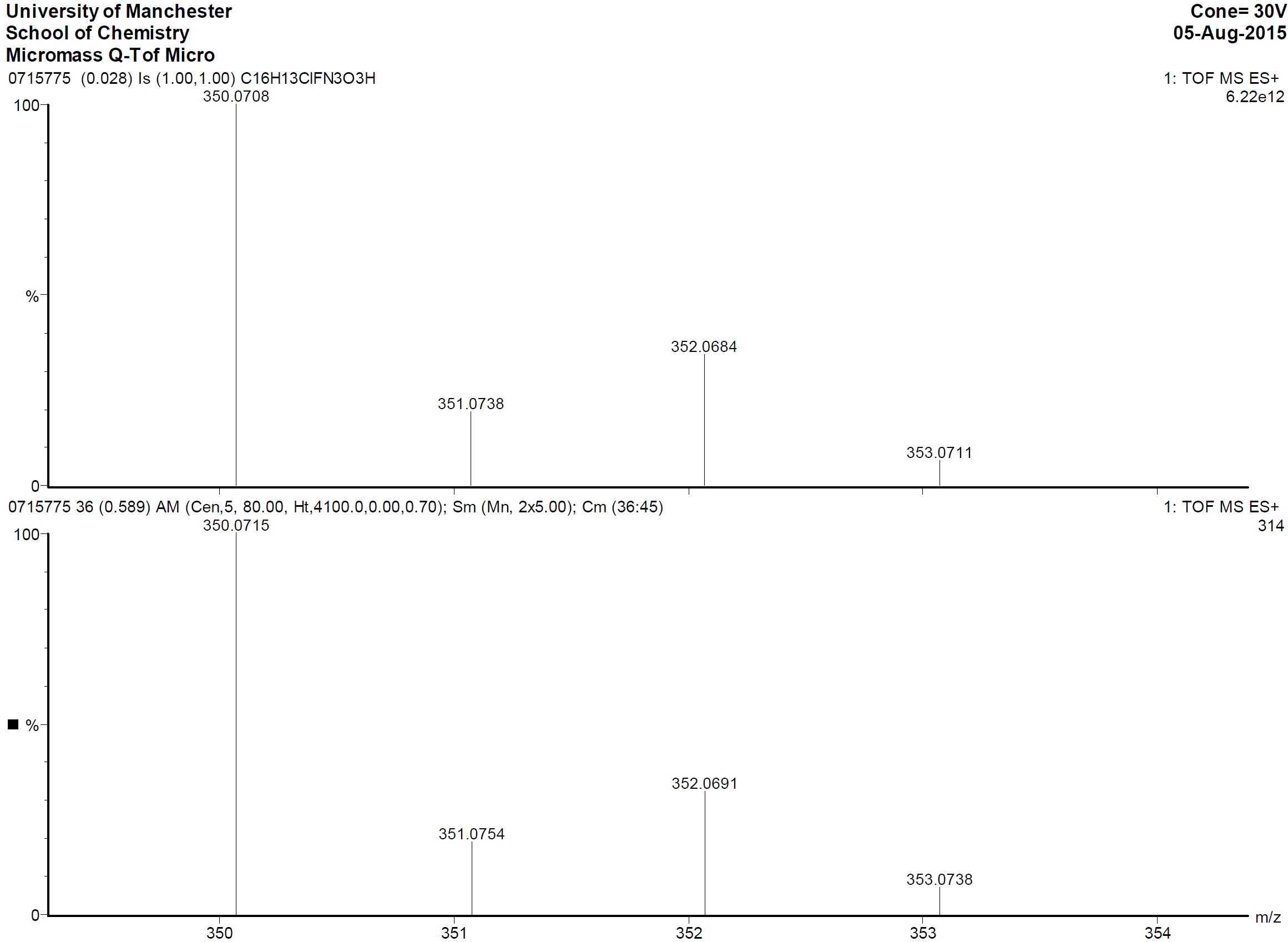


**Structure determination protocols**

*Production of RET-KD crystals containing* ***6***

GST-tagged recombinant RET kinase domain RET-KD (residues 705–1013) was expressed in Sf9 cells using a recombinant baculovirus and purified through a GST affinity tag as described previously.[^1^](#_ENREF_1) Residues 827 to 840 of the kinase insert region were omitted from the construct. **6** was introduced into the protein solution at a ratio of 3:1 before concentration. The protein–inhibitor complex was then concentrated to 4.5 mg/ml in crystallisation buffer (20 mM Tris pH 8, 100 mM NaCl, 1 mM DTT and 1 mM EDTA). Crystals were grown at 16 °C in sitting drops containing 3.4 M sodium formate, and 0.1 M sodium acetate pH 4.4. All crystals were harvested directly into the cryoprotectant oil, perfluoro-polyether (Hampton Research) and flash-frozen in liquid nitrogen.

*Data collection and structure solution*

Data were collected at the Diamond synchrotron beamline I03 as shown in Table S1 and processed using standard data integration and scaling software. The crystals belong to space group P4_3_2_1_2 with a single molecule in the asymmetric unit (Table S1). The structure was solved by molecular replacement using phosphorylated RET-KD-P protein (PDB code 2IVT) as a search model and omitting flexible regions and phosphotyrosines. The structure was refined using Phenix.refine^[2](#_ENREF_2)^ and rebuilt using Coot.[^3^](#_ENREF_3) The inhibitor library was generated with the ACEDRG (beta version from CCP4). Electron density for **6** and the RET kinase domain are of a good quality. The RET activation loop is phosphorylated on both Tyr900 (partial) and Tyr 905 sidechains. The structure was refined at 2.57Å to an R_work_ of 18.5% (and R_free_ 24.1%) shown in Table S1. The coordinates and structures factors have been deposited with PDB code 5AMN. Structure figures were generated with CCP4MQ.[^4^](#_ENREF_4)

**Table S1**. Data collection and refinement statistics for RET KD bound to compound **6**

**Data Collection**

Spacegroup P4_3_2_1_2

Cell Dimensions (/Å) 50.57, 50.57, 242.8

α, β, γ (/°) 90, 90, 90

Resolution (/Å) 60.7 - 2.57 (2.64 – 2.57)

R_pim_ (/%) 5.6 (24.3)

Completeness (/%) 99.9 (100)

Multiplicity 13.6 (14.5)

Wilson B 28.3

CC(1/2)       99.6     (75.9)

Mean(I)/sd   11.4     (3.3)

**Refinement** Total No. reflections (free) 10852 (551)

R_work_ / R_free_ (/%) 18.3 / 23.2

No. atoms

Protein 2207

Formate 30

Water 35

Inhibitor 37

B-factors

Protein 35.3

Formate 50.3

Water 37.6

Inhibitor 30.2

Rmsd

Bond lengths (/Å) 0.0131

Bond angles (/°) 1.202

Ramachandran

Favoured (/%) 96.8

Outliers (/%) 0.0

Clashscore 7.1

Rotamer Outliers 0.94

Parenthesis shows the value for the highest resolution shell

*Detailed structural description of* ***6*** *bound to RET KD*

Compound **6** has a distinctly non-planar arrangement, with a 55° angle between the dimethoxy-quinazolin-4-amine and phenol ring systems. The inhibitor hydrogen bonds to the RET hinge region (mainchain N-H of residue Ala807) with its quinazoline ring sandwiched between the sidechains of Leu730, Val738 and Ala756 on one side and Leu881 on the other. The phenol ring of **6** extends towards the RET “back” pocket and is wedged between the gatekeeper Val804 and Lys758. In comparison with the AMP-bound X-ray structure (PDB 2IVT), this displaces the Lys758 sidechain towards adopting an “active” conformation preserving the crucial salt bridge with Glu775. The phenolic hydroxyl group hydrogen bonds to the invariant Glu775 side chain, as well as the mainchain N-H of Asp 892 (Figure S1).


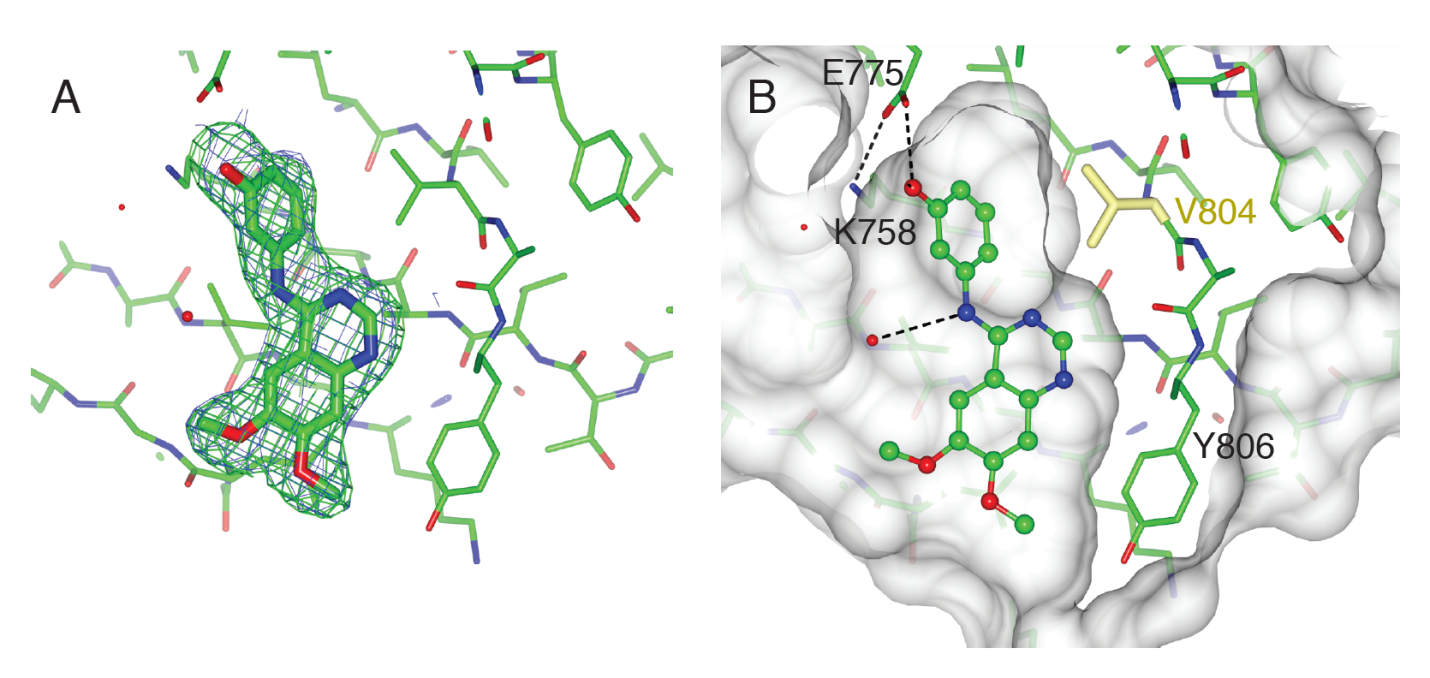


**Figure S1**. X-ray structure of RET complexed with compound **6**. (A) Compound **6** difference electron density contoured at 1.0σ (B) Surface representation of the binding site, highlighting hydrogen-bonding contacts between protein and compound **6**.

Binding of the inhibitor impacts on the RET kinase domain in several ways, compared to other published inhibitor complex structures,[^1^](#_ENREF_1) culminating in a space group change from monoclinic to a tetragonal cell. The glycine-rich loop (P-loop) adopts an "open" conformer resembling an ATP nucleotide-bound P-loop conformer, influenced by the compound’s non-planar shape. Sidechains Phe776, Leu800 and Leu802 on the αC helix patch are perturbed by inhibitor binding compared to a nucleotide-bound form of RET. A consequence of this is that the αN helix shifts by over 1Å relative to previous structures such as 2IVT. The loop containing residues Val804 to Tyr806 also shifts due to the presence of **6**.

**REFERENCES**

1. Knowles, P. P.; Murray-Rust, J.; Kjaer, S.; Scott, R. P.; Hanrahan, S.; Santoro, M.; Ibanez, C. F.; McDonald, N. Q. Structure and Chemical Inhibition of the RET Tyrosine Kinase Domain. *J. Biol. Chem.* **2006,** 281, 33577-33587.

2. Adams, P. D.; Afonine, P. V.; Bunkoczi, G.; Chen, V. B.; Davis, I. W.; Echols, N.; Headd, J. J.; Hung, L. W.; Kapral, G. J.; Grosse-Kunstleve, R. W.; McCoy, A. J.; Moriarty, N. W.; Oeffner, R.; Read, R. J.; Richardson, D. C.; Richardson, J. S.; Terwilliger, T. C.; Zwart, P. H. PHENIX: a comprehensive Python-based system for macromolecular structure solution. *Acta Crystallogr., Sect. D: Biol. Crystallogr.* **2010,** 66, 213-221.

3. Emsley, P.; Cowtan, K. Coot: model-building tools for molecular graphics. *Acta Crystallogr., Sect. D: Biol. Crystallogr.* **2004,** D60, 2126-2132.

4. McNicholas, S.; Potterton, E.; Wilson, K. S.; Noble, M. E. M. Presenting your structures: The CCP4mg molecular-graphics software. *Acta Crystallogr., Sect. D: Biol. Crystallogr.* **2011,** 67, 386-394.
